# Multispecies Populations of Methanotrophic *Methyloprofundus* and Cultivation of a Likely Dominant Species from the Iheya North Deep-Sea Hydrothermal Field

**DOI:** 10.1128/AEM.00758-21

**Published:** 2022-01-25

**Authors:** Hisako Hirayama, Yoshihiro Takaki, Mariko Abe, Hiroyuki Imachi, Tetsuro Ikuta, Junichi Miyazaki, Eiji Tasumi, Katsuyuki Uematsu, Akihiro Tame, Miwako Tsuda, Keiko Tanaka, Yohei Matsui, Hiromi Kayama Watanabe, Hiroyuki Yamamoto, Ken Takai

**Affiliations:** a Institute for Extra-cutting-edge Science and Technology Avant-garde Research (X-star), Japan Agency for Marine-Earth Science & Technology (JAMSTEC), Yokosuka, Japan; b Marine Biodiversity and Environmental Assessment Research Center (BioEnv), Research Institute for Global Change (RIGC), JAMSTEC, Yokosuka, Japan; c Marine Works Japan Ltd., Yokosuka, Japan; University of Michigan-Ann Arbor

**Keywords:** *Methyloprofundus*, methanotroph, deep-sea hydrothermal field, *Bathymodiolus*, symbiont, methane, methanol dehydrogenase

## Abstract

The *Methyloprofundus* clade is represented by uncultivated methanotrophic bacterial endosymbionts of deep-sea bathymodiolin mussels, but only a single free-living species has been cultivated to date. This study reveals the existence of free-living *Methyloprofundus* variants in the Iheya North deep-sea hydrothermal field in the mid-Okinawa Trough. A clade-targeted amplicon analysis of the particulate methane monooxygenase gene (*pmoA*) detected 647 amplicon sequence variants (ASVs) of the *Methyloprofundus* clade in microbial communities newly formed in *in situ* colonization systems. Such systems were deployed at colonies of bathymodiolin mussels and a galatheoid crab in diffuse-flow areas. These ASVs were classified into 161 species-like groups. The proportion of the species-like groups representing endosymbionts of mussels was unexpectedly low. A methanotrophic bacterium designated INp10, a likely dominant species in the *Methyloprofundus* population in this field, was enriched in a biofilm formed in a methane-fed cultivation system operated at 10°C. Genomic characterization with the gene transcription data set of INp10 from the biofilm suggested traits advantageous to niche competition in environments, such as mobility, chemotaxis, biofilm formation, offensive and defensive systems, and hypoxia tolerance. The notable metabolic traits that INp10 shares with some *Methyloprofundus* members are the use of lanthanide-dependent XoxF as the sole methanol dehydrogenase due to the absence of the canonical MxaFI, the glycolytic pathway using fructose-6-phosphate aldolase instead of fructose-1,6-bisphosphate aldolase, and the potential to perform partial denitrification from nitrate under oxygen-limited conditions. These findings help us better understand the ecological strategies of this possibly widespread marine-specific methanotrophic clade.

**IMPORTANCE** The Iheya North deep-sea hydrothermal field in the mid-Okinawa Trough is characterized by abundant methane derived from organic-rich sediments and diverse chemosynthetic animal species, including those harboring methanotrophic bacterial symbionts, such as bathymodiolin mussels Bathymodiolus japonicus and “*Bathymodiolus*” *platifrons* and a galatheoid crab, Shinkaia crosnieri. Symbiotic methanotrophs have attracted significant attention, and yet free-living methanotrophs in this environment have not been studied in detail. We focused on the free-living *Methyloprofundus* spp. that thrive in this hydrothermal field and identified an unexpectedly large number of species-like groups in this clade. Moreover, we enriched and characterized a methanotroph whose genome sequence indicated that it corresponds to a new species in the genus *Methyloprofundus*. This species might be a dominant member of the indigenous *Methyloprofundus* population. New information on free-living *Methyloprofundus* populations suggests that the hydrothermal field is a promising locale at which to investigate the adaptive capacity and associated genetic diversity of *Methyloprofundus* spp.

## INTRODUCTION

Methane-oxidizing, or methanotrophic, bacteria (MOB) are the final biological filters that reduce methane emissions into the atmosphere. This filter provided by MOB is thick and functional, particularly in marine ecosystems, and biological methane oxidation occurs even in low-methane environments (1–5). MOB require O_2_ to oxidize methane; however, their marine habitats may extend to hypoxic and anoxic environments (6–10), indicating that seamless bacterial methane oxidation occurs throughout marine ecosystems.

Most marine MOB belong to the order *Methylococcales* (*Gammaproteobacteria*), and some species have been cultivated and characterized (11–18). However, the majority of these species are still unidentified. The relative abundance of MOB is often low in environmental microbial communities, and *pmoA*, the gene encoding particulate methane monooxygenase (pMMO) subunit A, is frequently used as a gene marker for the detection of MOB (19–23). A recent comprehensive classification using large data sets of environmental *pmoA* sequences revealed habitat preferences among some *pmoA* clades (24). Marine-specific clades have been provisionally named “deep-sea clusters 1 to 5” (25). These clades include only a few, if any, cultivated members; thus, information on their genetic and physiological traits is limited. The *Methyloprofundus* clade (17) is one such group classified into both *pmoA* “deep-sea cluster 1” and 16S rRNA gene “Marine Methylotrophic Group 1” (22).

The *Methyloprofundus* clade is distinctive in that it includes many uncultivated endosymbionts of mussels of the subfamily Bathymodiolinae, which is one of the most successful animals in deep-sea chemosynthetic ecosystems (26). Bathymodiolin mussels harbor methanotrophs, thiotrophs, or both as primary bacterial endosymbionts in their gill tissues; methanotrophic endosymbionts are exclusively classified into the *Methyloprofundus* clade (27). Given that mussels have a strategy of environmental symbiont acquisition (28–30), it is reasonable to predict the presence of abundant free-living endosymbiont candidates (free-living pre-endosymbionts) around mussel colonies. Sequences of likely symbiotic origin were detected previously in biofilms and seawater near mussel colonies at deep-sea hydrothermal vents (28, 31). However, the methods used in those studies—traditional gene-cloning analysis and quantitative PCR—may lack resolution. Therefore, the abundance and activity of free-living pre-endosymbionts in the environment are still largely uncharacterized.

Meanwhile, *Methyloprofundus*-like sequences have been detected in various deep-sea samples, specifically those from cold seeps and hydrothermal fields. Their sources include sediments (19, 22, 32–35), microbial mats (32, 36, 37), an oil seep sponge (38), chimney fragments (39), and plume water (39–41). Notably, such sequences have been detected from oxygen minimum zones in the eastern tropical Pacific Ocean and a gulf in Costa Rica (9, 42). Nevertheless, the sole cultured species Methyloprofundus sedimenti WF1^T^ from deep-sea sediments was reported only in 2015 (17). *Methyloprofundus* spp. may be widespread in the marine environment, and they play a possibly significant role in the marine methane cycle. Despite this possibility, studies focusing on free-living *Methyloprofundus* spp. are limited.

The Iheya North deep-sea hydrothermal field in the mid-Okinawa Trough is characterized by methane-rich hydrothermal fluids with a maximum temperature of >300°C (43). Diverse faunal communities flourish in numerous diffuse-flow areas. The most prominent animal species here are Shinkaia crosnieri, a galatheoid crab that harbors both methanotrophic and thiotrophic bacterial epibionts; and two bathymodiolin mussels, namely, Bathymodiolus japonicus and “*Bathymodiolus*” *platifrons* that harbor methanotrophic endosymbionts (44–47). The genus classification of *Bathymodiolus* species is controversial; “*B.*” *platifrons* (hereafter, *B. platifrons*) is currently recognized as Gigantidas platifrons (48, 49). However, we use the traditional genus name *Bathymodiolus* for this species in the manuscript. A previous study in this field reported *pmoA* phylotypes from hydrothermal plumes, although their affiliations were not indicated (39). Using a BLAST search, we found that some of the phylotypes belong to the *Methyloprofundus* clade.

In light of previous findings, *Methyloprofundus* species are expected to flourish in the Iheya North deep-sea hydrothermal field. In this study, to clarify the presence of free-living *Methyloprofundus* spp. and delineate their populations in this field, we analyzed the newly formed microbial communities in *in situ* colonization systems (ISCSs) deployed at animal colonies. These systems collect free-living microbes on porous ceramic particles. In addition, we repeated cultivation experiments targeting methanotrophs with independent inocula from this field using a methane-fed flowthrough cultivation system that provides conditions closer to the natural habitat than closed batch cultivation using a rich medium. One of the purposes of the cultivations was to explore the culturability of mussel endosymbionts. No endosymbiont could be cultivated; however, we successfully enriched a *Methyloprofundus* species designated INp10. We characterized this species via complete genome sequencing and gene transcription profiling. *B. japonicus* and *B*. *platifrons* endosymbionts were also examined for a comparative analysis. The results are discussed in terms of ecological and physiological traits of free-living *Methyloprofundus* spp.

## RESULTS

### Study sites and observation of recovered ISCSs.

**FIG 1:**
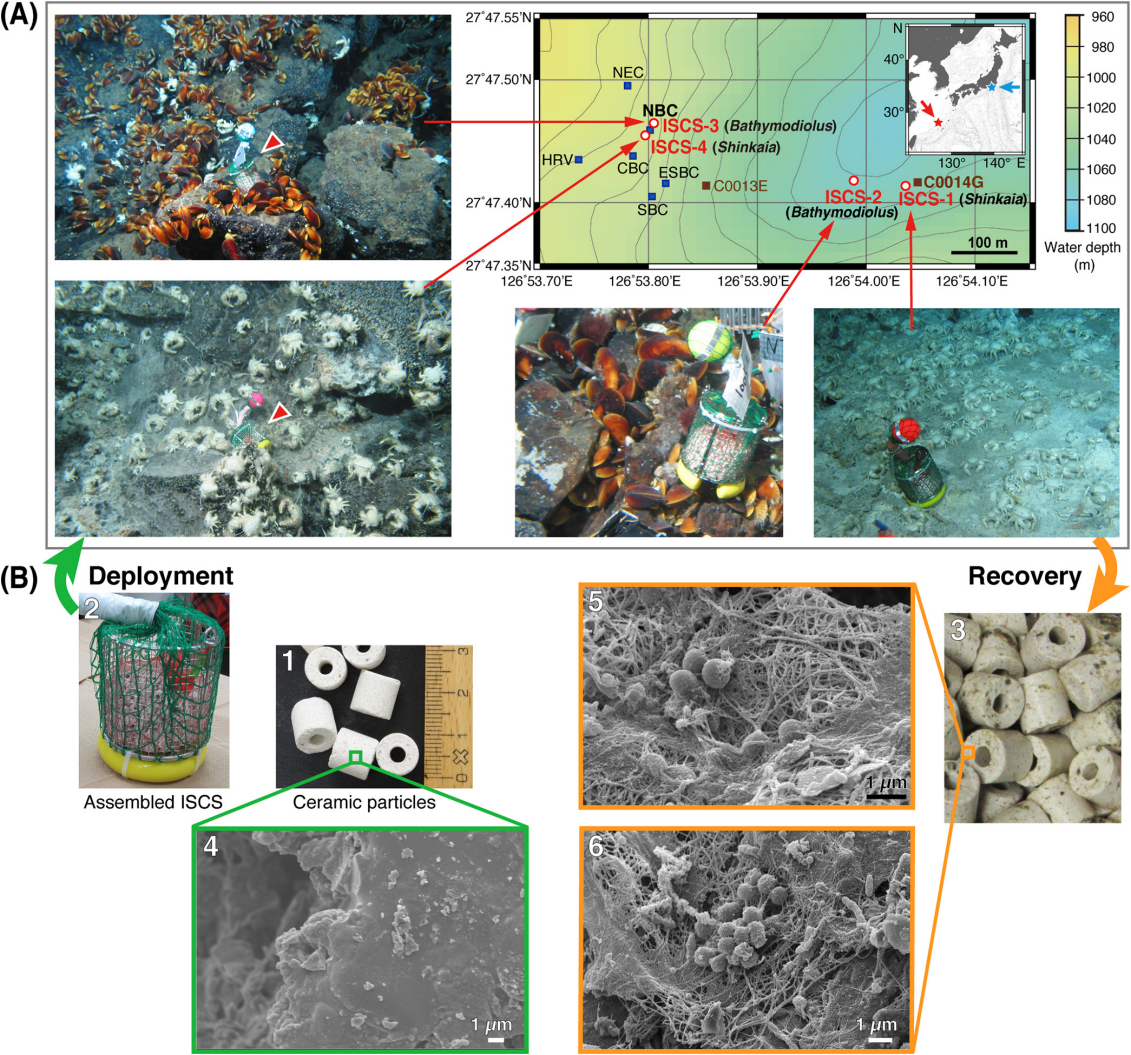
(A) Map of the Original site in the Iheya North deep-sea hydrothermal field, indicating the locations of the four ISCSs (red open circles) deployed at colonies of animals shown in parentheses for 2 months and the respective images of the deployed ISCSs. The active vent sites (blue squares; NBC, NEC, HRV, CBC, SBC, and ESBC) and the holes drilled during IODP expedition 331 (brown squares; C0014G and C0013E) are also indicated. The contour lines are shown at 10-m intervals. The map was generated with Generic Mapping Tools (GMT). The top right inset (based on map data from NOAA/NCEI) shows the locations of this field (red star) and a methane seep site in Sagami Bay (light-blue star) in the northwest Pacific Ocean. (B) Images of the ISCSs. (B-1) Ceramic particles before deployment. (B-2) The ISCS assembled for deployment. (B-3) Ceramic particles recovered after 2-month deployment. (B-4) An SEM image of a ceramic particle surface before deployment. (B-5 and B-6) SEM images of ceramic particle surfaces in the ISCS after recovery (ISCS-3 is shown as a representative).

Colonies of *B. japonicus*, *B*. *platifrons*, and *S. crosnieri*, which harbor methanotrophic symbionts, are bioindicators of the presence of methane (44, 47). This study used ISCSs to collect free-living microbes from these animal colonies. The porous ceramic particles used in ISCSs were expected to allow for microbial attachment and colonization. Four colonies were selected at the Original site (50) in the Iheya North deep-sea hydrothermal field in anticipation of the presence of different methanotrophs, and ISCSs were deployed there for 2 months (Fig. 1A, B, and B-2; Table 1). Visible hydrothermal diffuse flows were typically observed at *S. crosnieri* colonies but not at bathymodiolin mussel colonies. ISCS-1 was deployed at an *S. crosnieri* colony near Hole C0014G, an artificial hydrothermal vent drilled during the Integrated Ocean Drilling Program (IODP) Expedition 331 in 2010 (51). The emergence of diffuse flows after drilling enabled *S. crosnieri* to settle in this site (43). ISCS-2 was deployed at a mussel colony, around which small colonies of mussels and *S. crosnieri* were observed in patches on the rocky seafloor. The North Big Chimney (NBC) is an active hydrothermal mound that is >10 m high and is inhabited by numerous invertebrates (45, 46); ISCS-3 and ISCS-4 were deployed at the foot (mussel colony) and hillside (*S. crosnieri* colony), respectively, of the NBC.

**TABLE 1:** Description of the ISCS-deployed sites in the Original site in the Iheya North deep-sea hydrothermal field

Sample	Site characteristic	Environmental parameter data^*a*^						
		Depth (m)	Temp. (°C)	pH	DO (μM)	CH_4_ (μM)	H_2_S (μM)	H_2_ (μM)
ISCS-1	Colony of *S. crosnieri* near C0014G borehole	1,058	4.3–4.8 (4.5)	n.d.	80.6–96.6 (91.9)	n.d.	29.3–92.6 (62.6)	5.7–19.4 (12.9)
ISCS-2	Colony of *Bathymodiolus* mussels between C0013 and C0014 sites	1,061	4.1–4.3 (4.2)	n.d.	86.5–93.8 (89.3)	n.d.	0–103 (48.4)	0–1.4 (0.2)
ISCS-3	Colony of *Bathymodiolus* mussels at the foot of the NBC mound	994	4.3 (4.3)	7.2–7.6	93.2–93.4 (93.3)	8.8–10.1 (9.8)	<1–40 (12.8)	0.4–1.5 (0.8)
ISCS-4	Colony of *S. crosnieri* at the hillside of the NBC mound	986	5.1–8.7 (5.7)	7.5	82.5–95.2 (89.4)	19.8–23.0 (22.2)	45.6–118 (73.8)	7.3–11.8 (8.2)

a Average values are shown in parentheses. DO, dissolved oxygen. n.d., not determined.

**FIG 2:**
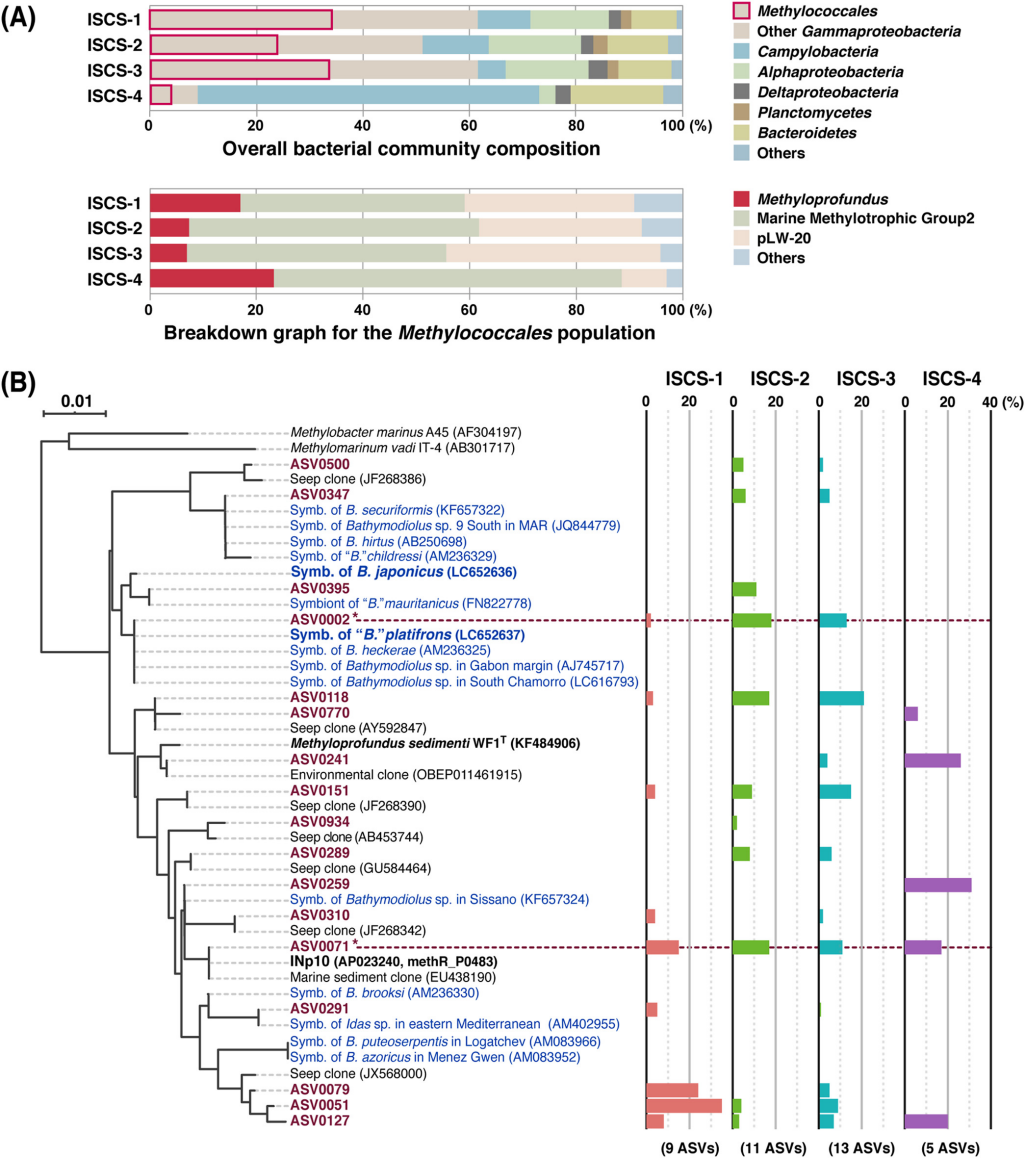
ISCS community based on the 16S rRNA gene amplicon analysis. (A) The top graph represents overall bacterial community composition showing the dominant phyla, classes, and the order *Methylococcales* (highlighted). The bottom graph shows a breakdown graph for the *Methylococcales* population by taxonomic groups suggested in the SILVA database. (B) NJ tree and relative abundance of 17 ASVs_-16S_ within the *Methyloprofundus* population in each ISCS. Reference sequences are from symbionts of bathymodiolin mussels (shown with blue characters), environmental clones showing high similarity to ASVs_-16S_ by BLAST search, *M. sedimenti* WF1^T^, and *Methyloprofundus* sp. INp10 enriched in this study. Accession numbers are shown in parentheses; for INp10, the gene locus tag is shown along with the genome accession number. The ASVs_-16S_ with identical sequences to the *B*. *platifrons* symbiont and INp10 are marked with asterisks. The number of ASVs_-16S_ detected in each ISCS is shown at the bottom of the graph in parentheses. MAR, Mid-Atlantic Ridge.

**FIG 3:**
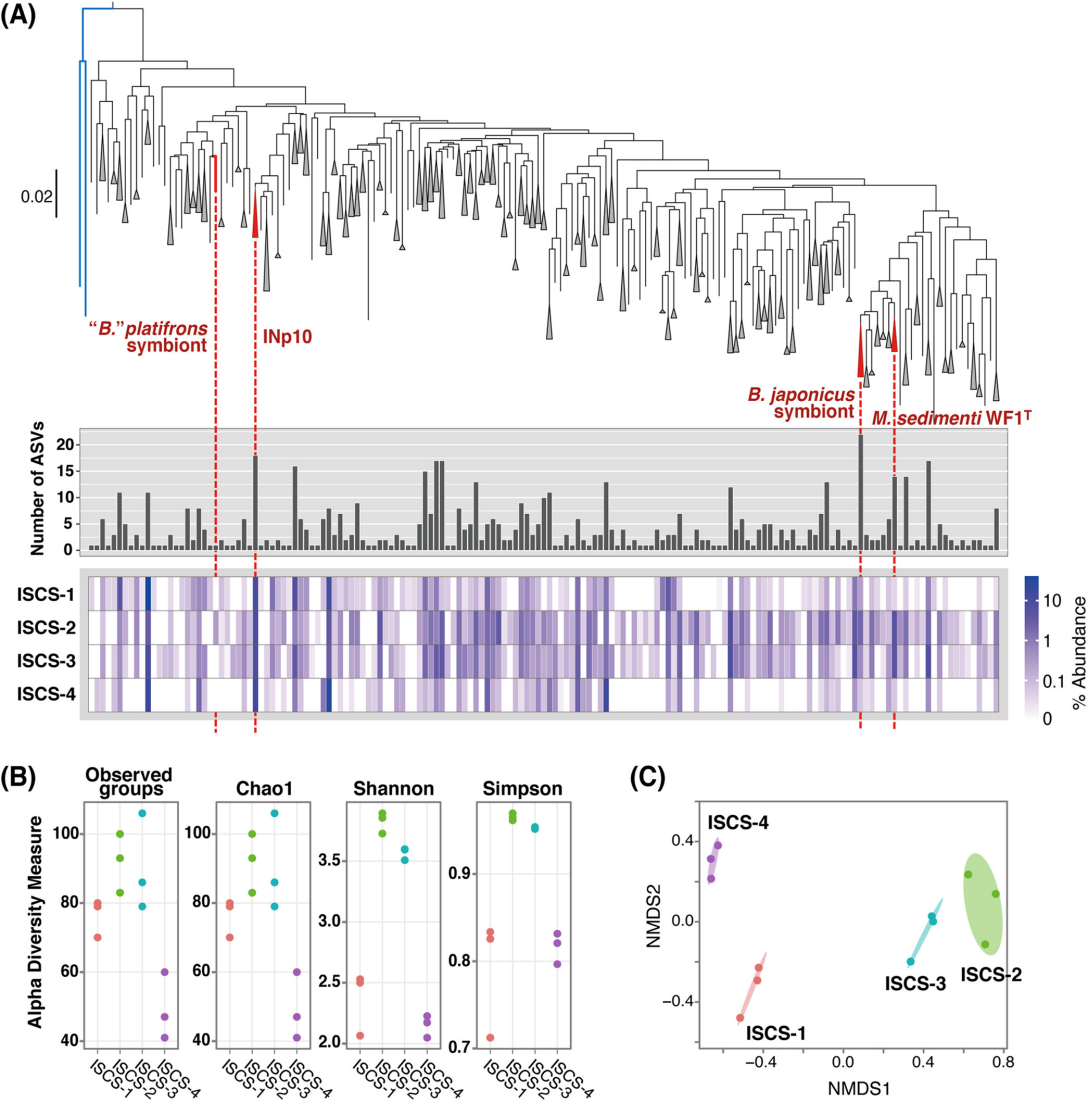
*Methyloprofundus* populations in the ISCSs detected by *pmoA* amplicon analysis. (A) An NJ tree of species-like groups (top), total number of ASVs_-pmoA_ included in each species-like group (middle), and composite heatmap showing the species-like group compositions (averaged over triplicate samples in each ISCS; for individual heatmap, see Fig. S2) (bottom). Reference *pmoA* sequences are from a *B. japonicus* endosymbiont (GenBank accession no. LC652638), *B*. *platifrons* endosymbiont (LC652639), *M. sedimenti* WF1^T^ (KF484908), and *Methyloprofundus* sp. INp10 enriched in this study (AP023240; locus tag, methR_P0112). An outgroup in the tree is the MMG2 clade sequences obtained in this study (branches in blue lines). (B) Alpha diversity (Chao1, Shannon, and Simpson diversity indices) of the species-like group population in each sample. (C) NMDS plot (Bray-Curtis dissimilarity; stress, 0.0196) showing the similarity of the species-like group populations. Ellipses indicate 95% confidence intervals for the populations from the respective ISCSs.

**FIG 4:**
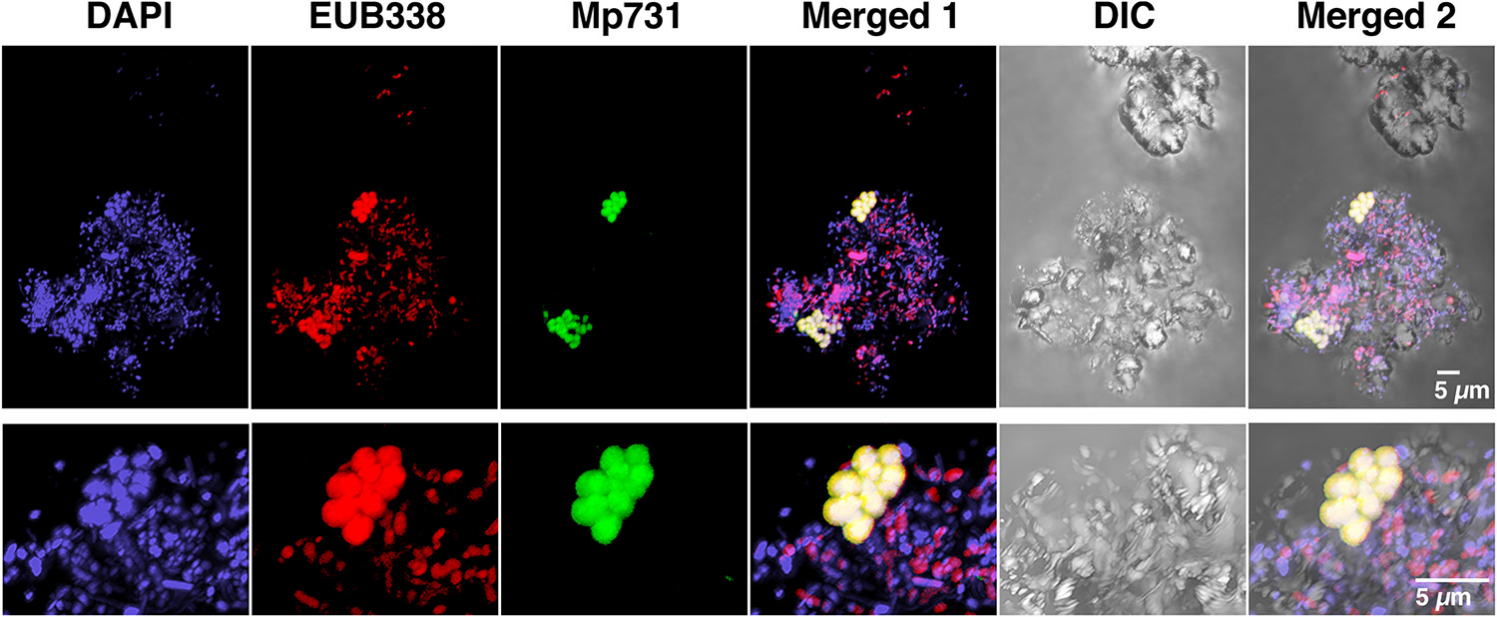
Whole-cell FISH images of *Methyloprofundus*-like bacteria colonized on a ceramic particle of the ISCS. ISCS-4 is shown as a representative. The Mp731 probe was designed to detect the *Methyloprofundus* clade. The three images on the left, with 4′,6-diamidino-2-phenylindole (DAPI) staining and FISH with the EUB338 and Mp731 probes, were merged and labeled “merged 1.” In addition, the image by differential interference contrast (DIC) microscopy was further merged and labeled “merged 2.” The images in the top row are partially enlarged and shown in the bottom row. The MP731 and Eub338 probes were labeled with Alexa Fluor 488 and 555, respectively.

**FIG 5:**
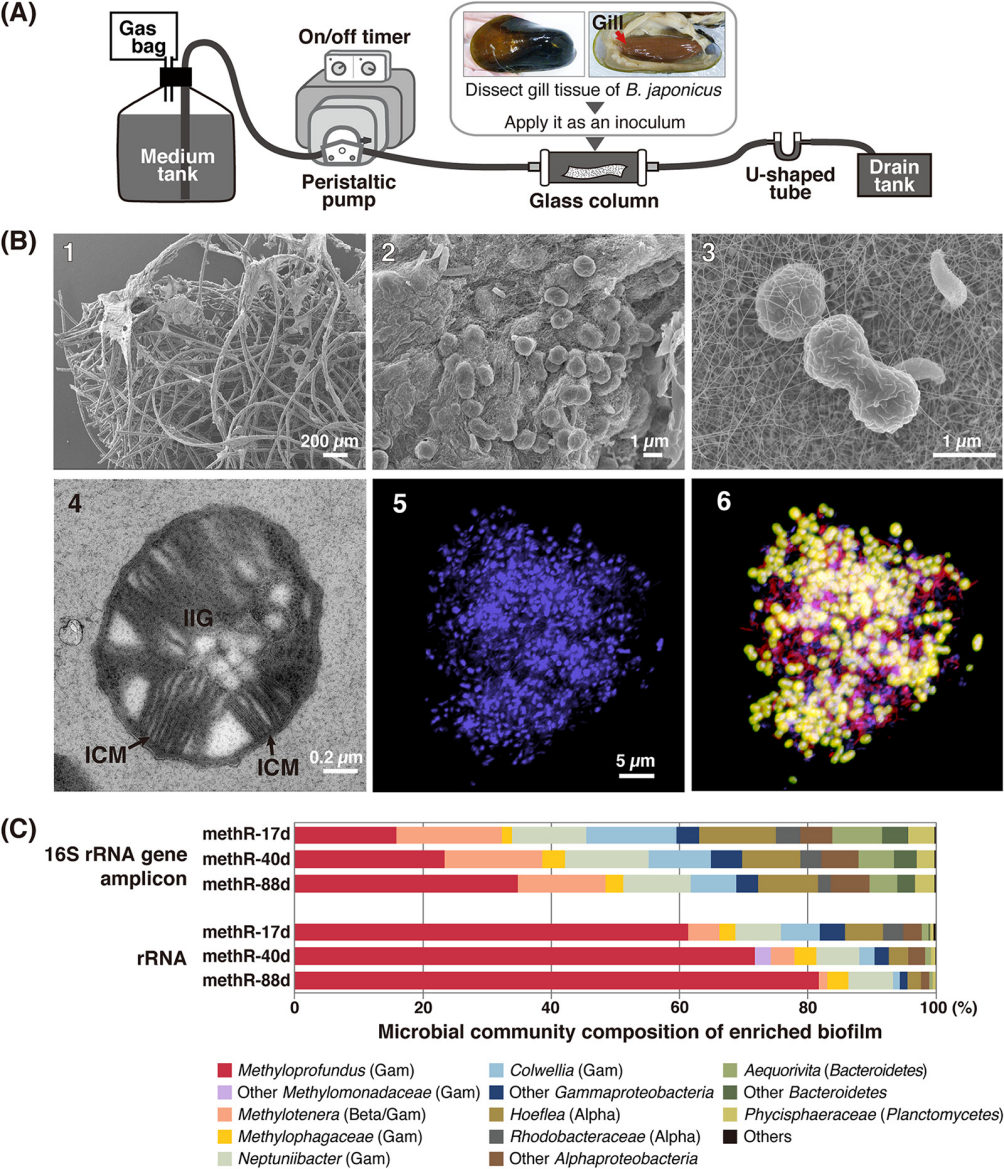
(A) A schematic drawing of the continuous flowthrough cultivation system fed with methane. The medium containing dissolved methane and DO was supplied to the system by a peristaltic pump that worked periodically with an on-off timer. (B) Images of biofilms grown in the cultivation system. The growth period after subculturing is shown in parentheses. (B-1 and B-2) SEM micrographs of biofilms (4 months); the biofilm attached on fibers of polyethylene nonwoven fabric (B-1) and methanotroph-like coccoid cells (B-2). (B-3) A SEM micrograph of methanotroph-like cells in a thin biofilm (21 days). (B-4) A TEM micrograph of a cell with intracytoplasmic membranes (ICMs) typical for gammaproteobacterial methanotrophs and intracellular inclusions of granules (IIG) (1 month). (B-5 and B-6) Whole-cell FISH images of biofilm (1 month); DAPI-stained image (B-5) and merged image of the Mp731 probe (green), EUB338 probe (red), and DAPI-stained images, where the triple-stained cells look green-yellow (B-6). (C) Microbial community compositions of biofilms grown for 17, 40, and 80 days (methR-17d, methR-40d, and methR-88d, respectively) based on 16S rRNA gene amplicon sequences and rRNA sequences. The sequences were grouped based on the SILVA classification. The genus- and family-level taxa that accounted for >3% of relative abundances in either of the six samples are shown by those names. Gam, *Gammaproteobacteria*; Beta, *Betaproteobacteria*; Alpha, *Alphaproteobacteria*.

**FIG 6:**
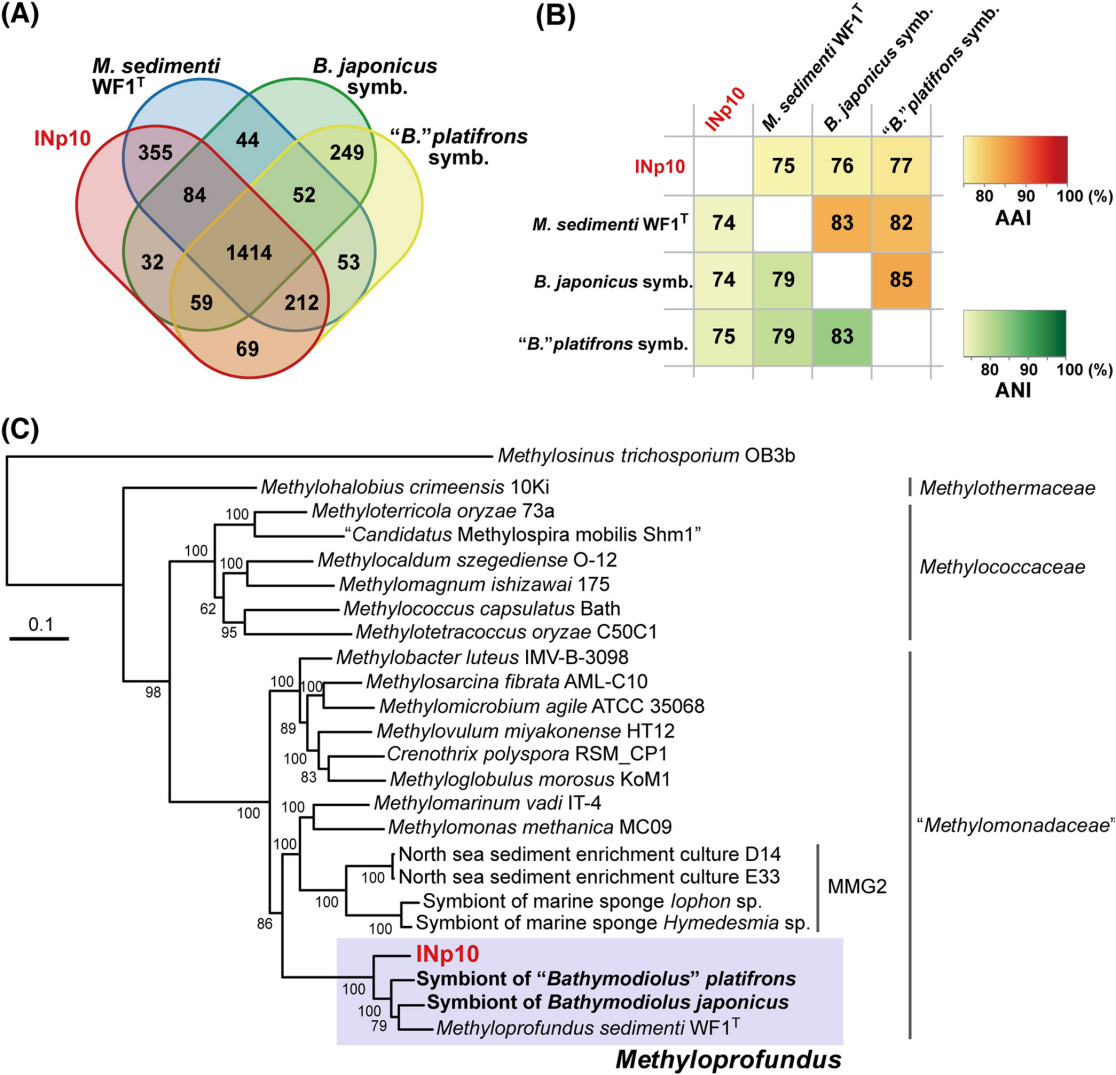
Genomic comparison among the four methanotrophs of *Methyloprofundus*, as follows: INp10, *B. japonicus* and *B*. *platifrons* endosymbionts, and *M. sedimenti* WF1^T^. (A) Venn diagram showing the number of shared protein-coding genes between the genomes. (B) Heatmap showing the ANI and AAI between the genomes. (C) Phylogenomic tree showing the positions of *Methyloprofundus* species and reference strains of the order *Methylococcales* with available high-quality genomes. The concatenated amino acid sequences of 30 single-copy marker genes were used in the analysis. The sequence of Methylosinus trichosporium OB3b was used as the outgroup. The confidence of branch points was estimated by 100 bootstrap replications and indicated at each point. Scale bar represents the number of substitutions per position.

Environmental conditions at the ISCS sites were observed (Table 1). Some measurements varied over the course of several minutes, probably due to the steep gradients formed by the immediate mixing of hydrothermal fluids and ambient seawater. Molecular hydrogen (H_2_) concentrations indicated that *S. crosnieri* colonies are more affected by hydrothermal fluids than mussel colonies. This finding was consistent through visual observations of the colonies as well as by signs of heat exposure found only in ISCS-1 and ISCS-4; the polyvinyl chloride coating of sinkers was partially melted, indicating that the bottoms of the ISCSs were at least partly and temporarily exposed to water at temperatures of ≥90°C. Ceramic particles from the recovered ISCSs were brown and grimy (Fig. 1B-3). Scanning electron microscopy (SEM) of ISCS-3 as a representative sample indicated the formation of thin biofilms (Fig. 1B-4 to B-6) and clusters of similarly shaped microbial cells (Fig. 1B-6) in patches on the particles; these findings indicate *in situ* microbial growth. The thickness of the biofilm was up to several micrometers in the range observed via focused ion beam (FIB)-SEM (see Fig. S1 in the supplemental material).

### ISCS communities.

### (i) 16S rRNA gene analysis.

The 16S rRNA gene amplicon analysis was employed for an overall perspective of total bacterial communities and MOB taxa from ISCSs. This analysis detected members of the order *Methylococcales* but no other known MOB taxa. The relative abundance of *Methylococcales* was 24% to 34% for ISCS-1, ISCS-2, and ISCS-3 and represented about one-half of all *Gammaproteobacteria*. Contrarily, in ISCS-4, *Methylococcales* accounted for only 4% of the population, and the class *Campylobacteria* (formerly *Epsilonproteobacteria*) (52) was dominant (65%) (Fig. 2A, top graph). *Sulfurimonas* and *Sulfurovum* species were the dominant members of *Campylobacteria* across all ISCSs (data not shown). The taxonomic breakdown of *Methylococcales* revealed three dominant clades, namely, *Methyloprofundus*, Marine Methylotrophic Group 2 (MMG2), and pLW-20, constituting 91% to 97% of the *Methylococcales* population (Fig. 2A, bottom graph). The *Methyloprofundus* clade comprised 7% to 24% of the *Methylococcales* population and 1% to 6% of the whole bacterial community.

A total of 17 amplicon sequence variants (ASVs) of the 16S rRNA gene (ASVs_-16S_) were assigned to the *Methyloprofundus* clade; 5 to 13 ASVs_-16S_ were detected in each library (Fig. 2B). Five ASVs_-16S_ were identical to one or more endosymbiont sequences from bathymodiolin mussels in the database, including *B*. *platifrons* in this field (ASV0002); no ASV_-16S_ representing the *B. japonicus* endosymbiont was detected. A total of 12 ASVs_-16S_ were closest to environmental clones, which were mostly from seeps. Notably, ASV_-16S_ (ASV0071), representing INp10, an enriched *Methyloprofundus* species described below, was detected in all ISCSs, accounting for 11% to 17% of the respective *Methyloprofundus* populations. Furthermore, the analysis did not display a clear species-level resolution. For example, the amplified region of a sequence from the *B*. *platifron*s endosymbiont (GenBank accession no. LC652637) was identical to sequences with the accession numbers AM236325, AJ745717, and LC616793 that are from endosymbionts of other bathymodioline mussels (Fig. 2B). However, a comparison of nearly full-length sequences revealed that the *B*. *platifron*s endosymbiont sequence was <97.5% similar to the others. This similarity is well below the 98.65% that was proposed as a species boundary (53).

### (ii) *pmoA* analysis of *Methyloprofundus* populations.

A more in-depth analysis of the *Methyloprofundus* populations was conducted on biological triplicates from each ISCS using *pmoA* amplicon sequencing with new primers. Of all the amplicons generated using these primers, 17% to 75% were assigned to the *Methyloprofundus* clade in each sample, whereas the rest were assigned to MMG2 or could not be clearly assigned to the known clades. A total of 647 ASVs of *pmoA* (ASVs_-pmoA_) were generated from the 50,000 reads assigned to the *Methyloprofundus* clade in each sample. The maximum nucleotide sequence difference between ASV_-pmoA_ and *pmoA* of the type species *M. sedimenti* WF1^T^ was 15.7%. For reference, these 647 ASVs_-pmoA_ were translated to amino acid sequences and integrated into 175 sequences. All of the translated sequences contained the conserved domain of pMMO (Pfam PF02461).

To delineate ASVs_-pmoA_ as a species-level population of the *Methyloprofundus* clade, ASVs_-pmoA_ were classified following the reported numerical criterion for species identity. The 647 ASVs_-pmoA_ were grouped on a neighbor-joining (NJ) tree adopting a distance threshold of 0.04 (Jukes-Cantor distance), corresponding to the 4% cutoff suggested by Knief (24) for a *pmoA* nucleotide sequence to distinguish between species. A total of 161 species-like groups were generated (Fig. 3A). These groups are not equivalent to taxonomic species but do suggest the existence of taxonomic species with similar sequences. The composite heatmap shows the averaged species-like group composition for each ISCS (see Fig. S2 in the supplemental material that shows individual heatmaps of the samples). Of 161 species-like groups, 118 were detected in multiple ISCSs, of which 52 were detected in all ISCSs, whereas 43 were detected only in a single ISCS, of which 29 were only in a single sample. Species-like groups with higher relative abundance tended to contain a higher number of ASVs_-pmoA_.

The species-like groups representing the endosymbionts of bathymodiolin mussels living in this field were identified. The endosymbiont sequences in the database were used as references. The group representing the *B. japonicus* endosymbiont was detected in all ISCSs (0.09% to 2.7% of the *Methyloprofundus* populations). This group contained 22 ASVs_-pmoA_, which is the maximum number in a single species-like group, but curiously, none of these ASVs_-pmoA_ was identical to the endosymbiont sequence. Conversely, the ASV_-pmoA_ identical to the *pmoA* of the *B*. *platifrons* endosymbiont was not grouped with others and was detected only in ISCS-2 (0.08% to 0.68%). The species-like group representing INp10 was a dominant group detected in all replicate samples from all ISCSs (Fig. S2). This group accounted for 7.0% to 17.2% and contained 18 ASVs_-pmoA_, including the one identical to the *pmoA* of INp10. Incidentally, these results were not entirely consistent with those of 16S rRNA gene amplicon analysis; for example, the presence/absence and relative abundance of ASVs representing the mussel endosymbionts and *M. sedimenti* WF1^T^ differed between the analyses.

### (iii) Alpha and beta diversity of *Methyloprofundus* populations.

Species-like group populations of the *Methyloprofundus* clade were analyzed for alpha and beta diversity. The estimated alpha diversity indices (Chao1, Shannon, and Simpson) were generally higher in ISCS-2 and ISCS-3 in terms of both richness and evenness (Fig. 3B; see Fig. S3 in the supplemental material that shows rank-abundance curves). The Kruskal-Wallis test of variance showed significant differences in all diversity indices among ISCSs (*P < *0.05), but *post hoc* testing indicated a significant difference only for the Shannon index between ISCS-2 and ISCS-4 (Bonferroni-adjusted, *P < *0.0167). Nonmetric multidimensional scaling (NMDS) ordination based on the Bray-Curtis dissimilarities showed a separation of samples by the ISCS source (Fig. 3C). A test for a significant clustering of samples was not performed as the number of samples per ISCS was only three. Two samples from the same ISCS and those from the different ISCSs showed roughly equal point-to-point distances in some cases between ISCS-2 and ISCS-3. Thus, the free-living *Methyloprofundus* populations associated with the two bathymodiolin mussel colonies appeared similar. Contrarily, each cluster of samples of ISCS-1 and ISCS-4 was isolated, indicating that the *Methyloprofundus* populations associated with *S. crosnieri* colonies had distinct structures.

### (iv) Fluorescence *in situ* hybridization for detecting *Methyloprofundus*.

Fluorescence *in situ* hybridization (FISH) analysis successfully identified *Methyloprofundus*-like cells from ISCSs (Fig. 4; see Fig. S4 in the supplemental material). Among the many bacterial cells hybridized with the universal bacterial probe Eub338, the Mp731 probe designed for *Methyloprofundus* spp. hybridized coccoid to elongated coccoid cells. These Mp731 probe-positive cells were often found as clusters, indicating that they multiplied in ISCSs as inferred from SEM observations (Fig. 1B).

### Enrichment of methanotrophs.

Methanotrophs from the Iheya North hydrothermal field were cultivated. On performing cultivation experiments with different inocula, we found a single methanotroph that was highly enriched from the gill tissue of *B. japonicus* from the NBC in this field. Cultivation was initiated at 10°C and different pH values (6.8 and 7.5) using a continuous flow-through system with an initial expectation of growth of the endosymbiont (Fig. 5A). The dissolved methane and oxygen (DO) concentrations in the medium were 390 to 900 μM and 130 to 170 μM, respectively. Microbial growth was observed at pH 7.5 after 2 weeks. Cultivation at pH 6.8 exhibited poor growth and was terminated after 5 months. Biofilm formation was observed as growth progressed (Fig. 5B-1 to B-3).

Microbial taxonomic composition of biofilms sampled at 17, 40, and 88 days after subculturing of mature biofilms (designated methR-17d, 40d, and 88d) was assessed based on both 16S rRNA gene amplicons and rRNA assemblages (Fig. 5C). The genus *Methyloprofundus* was the exclusive methanotrophic taxon across biofilms. Amplicon analysis detected a single ASV assigned to the genus *Methyloprofundus*, with relative abundances of 15.9%, 23.4%, and 34.9%. This *Methyloprofundus* sp. was designated INp10. Higher *Methyloprofundus* proportions were detected in rRNA assemblages and biofilms grown for longer periods. The methylotrophic taxa *Methylotenera* and *Methylophagaceae* were also detected. The metabolite profiles of culture supernatants showed 46 to 123 μM methanol, 7 to 12 μM formate, and 31 to 54 μM acetate in three independent samples collected 3 months after subculturing. The biofilm doubling time estimated via protein content increase was 5.2 days on average at the initial stage of biofilm formation.

Transmission electron microscopy (TEM) and FISH analysis on the biofilms confirmed the presence of gammaproteobacterial methanotrophs. TEM detected many coccoid to elongated coccoid cells (1.0 to 1.4 μm in size) with intracytoplasmic membranes. (Fig. 5B-4). In FISH analysis, the Mp731 probe hybridized these cells, but the thin rod-shaped cells were hybridized only with the Eub338 probe (Fig. 5B-5 and B-6). Cocci and elongated cocci were most likely to be *Methyloprofundus* methanotrophs.

Two additional independent methanotrophic enrichment cultures were obtained at 5°C under almost the same cultivation conditions as above from the following inocula: (i) gill tissue of *B. japonicus* collected in the same field but a different cruise and (ii) a mixture of ISCS-2 and ISCS-3. These cultures included *Methyloprofundus* species with 16S rRNA gene sequences identical to INp10 in the aforementioned culture. Thus, further analysis was confined to the aforementioned culture.

### Genome reconstructions and phylogenomic comparison of *Methyloprofundus* species.

To obtain the genomic data of *Methyloprofundus* sp. INp10 grown in biofilms, a metagenomic analysis of the methR-40d biofilm was conducted using the PacBio Sequel platform, which enables high sequence contiguity. A complete genome of INp10 was reconstructed from the metagenomic assembly as two circularized sequences composed of a chromosome (4.39 Mb) and a plasmid (42 kb), with an average genome coverage of 483×, and 99.7% completeness and 1.21% contamination based on the presence or absence of lineage-specific single-copy marker genes (see Table S1 in the supplemental material). This study referred to the following three genomes for comparison: *M. sedimenti* WF1^T^ (54) from the NCBI database and *B. japonicus* and *B*. *platifrons* endosymbionts from this study (Table 2; Table S1). The reference endosymbiont genome sequences were analyzed using mussels collected from the Sagami Bay seep (Fig. 1A) to ensure consistency with transcriptomic analysis; this analysis is constrained by the availability of high-quality RNA (see the next section). Regardless of the distance (>1,400 km) between Sagami Bay and the Iheya North field, high gene flow between the respective mussel populations has been estimated (55). We observed that the endosymbionts of each mussel species belong to the same species-level taxon regardless of their habitats, based on both the 16S rRNA gene and *pmoA* similarities (99.6% to 100% sequence identity; detailed data not shown). The draft genome sequences obtained for both endosymbionts are composite sequences that reflect high strain-level heterogeneity. These genome sequences are characterized by massive insertion sequence elements (>20% of each sequence), but most transposase genes were disrupted pseudogenes.

**TABLE 2:** The presence and absence of genes for the central metabolisms and some selected characteristics in the genomes of the four *Methyloprofundus* species

Characteristic	Data^*a*^ for:			
	INp10	*M. sedimenti* WF1^T^	Endosymbiont of *B. japonicus*	Endosymbiont of *B. platifrons*
Lifestyle	Free-living	Free-living	Symbiosis	Symbiosis
Genome assembly status	Complete	Draft	Draft	Draft
Genome size (Mb)^*b*^	4.39 (+0.04)	4.29	4.97	6.59
G+C content (mol%)	39.9	41.0	41.8	40.3
No. of protein-coding genes	3,774	3,699	2,869	4,160
Gene pathway				
Methane metabolism and respiration related				
Particulate methane monooxygenase (*pmoCAB*)^*c*^	+ (1)	+ (1)	+ (1)	+ (1)
Membrane-bound monooxygenase (*pxmABC*)	–	–	–	–
MxaFI-type methanol dehydrogenase (*mxaFI*)^*b*^	–	+ (1)	–	–
XoxF-type methanol dehydrogenase (*xoxF*)^*b*^	+ (1)	+ (1)	+ (1)	+ (1)
** **Aerobic respiratory complexes (I, II, III, and IV)	+	+	+	+
** **Cytochrome *d* ubiquinol oxidase (*cydAB*)	+	+	–	–
** **Hemerythrin	+	+	–	+
Central carbon metabolism				
** **Ribulose monophosphate pathway	+	+	+	+
** **Serine pathway	Incomplete	Incomplete	Incomplete	Incomplete
** **Entner-Doudoroff (ED) pathway	+	+	Incomplete	+
** **Embden-Meyerhof-Parnas (EMP) pathway	+	+	+	+
** **6-Phosphofructokinase (*pfk*)	–	–	–	–
** **Fructose-1,6-bisphosphate aldolase (*fba*)	–	–	–	–
** **Fructose-6-phosphate aldolase (*fsaA*)	+	+	+	+
** **Dihydroxyacetone kinase (*dhaKL*)	+	+	+	+
** **TCA cycle	+	+	+	+
Nitrogen metabolism				
** **Nitrate reductase (*narGHI*)	+	+	+	+
** **Nitrite reductase, copper containing (NO-forming) (*nirK*)	+	+	+	+
** **Nitrite reductase, cytochrome cd1 (NO-forming) (*nirS*)	+	+	–	+
** **Nitrite reductase (NADH) (nitrite to ammonia) (*nirBD*)	+	+	+	+
** **Nitric oxide reductase (*norBC*)	+	+	–	–
** **Nitric oxide reductase (*norZ*)	–	+	–	–
** **Hydroxylamine dehydrogenase (*hao*)	–	–	–	–
** **Hydroxylamine reductase (*hcp*)	+	+	+	+
** **Ammonium transporter (*amt*)	+	+	+	+
** **Nitrate/nitrite transporter (*narK*)	+	+	+	+
** **Nitrogenase (*nifHDK*)	–	+	–	–
Carbohydrate synthesis				
Glycogen synthesis (*glgCAB*)	+	+	+	+
Cellulose synthesis (*bcsABCZ*)	+	–	–	–
Sucrose synthesis	–	+	–	–
Mobility, chemotaxis, secretion, antibiotics				
** **Flagellum	+	–	–	–
** **Pilus assembly	+	+	–	–
** **Chemotaxis system^*d*^	+ (3)	–	–	–
** **Type II secretion system	+	+	+	+
** **Type VI secretion system	+	–	–	–
** **Enediyne biosynthesis	+	–	–	–
No. of toxin-antitoxins	57	13	20	73
No. of insertion sequence elements^*e*^	185 (120)	92 (66)	1,303 (300)	1,847 (685)
Reference	This study	54	This study	This study

a +, presence; –, absence.

b Plasmid size is shown in parentheses.

c The number of (sets of) genes is shown in parentheses.

d The number of sets of core genes (*cheABWY*) is shown in parentheses.

e The number of complete insertion sequence elements or complete transposase genes is shown in parentheses. For *M. sedimenti* WF1^T^, the number of transposase genes is shown.

A total of 3,774 protein-coding genes were identified in the INp10 genome. Approximately 60% of genes were shared with one or more samples (Fig. 6A). The average nucleotide identity (ANI) among the four genomes was 74% to 83%, which was lower than the proposed 95% to 96% ANI threshold for species delineation (Fig. 6B) (56, 57). Moreover, the average amino acid identity (AAI) of 75% to 85% was below the 95% AAI threshold for species delineation but was within the range for the same genus (≥65%) (58). In the phylogenomic tree based on 30 single-copy marker genes (see Table S2 in the supplemental material), the 4 *Methyloprofundus* members formed a monophyletic group but were not separated by lifestyles (free-living or symbiotic) (Fig. 6C). INp10 thus represents a new species in the genus *Methyloprofundus*. This designation is supported by the 16S rRNA gene sequence comparison of INp10 with *M. sedimenti* WF1^T^ (98.2% identity) and methanotrophic endosymbionts from bathymodiolin mussels (97.1% to 98.1%) referenced in Fig. 2B. These values are less than the proposed species boundary (98.65%) (53).

### Characterization of INp10 by genomic and transcriptomic information.

**FIG 7:**
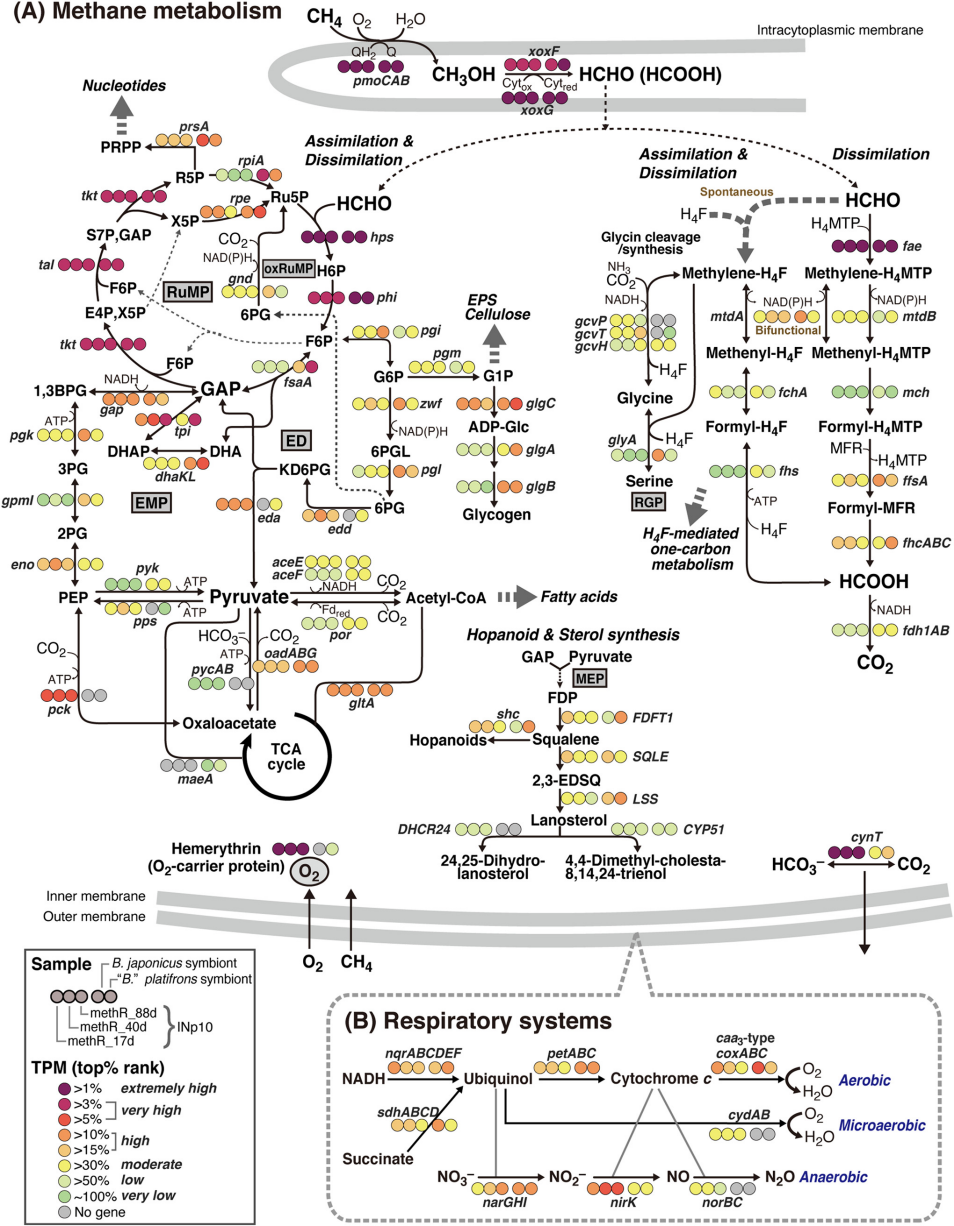
Metabolic pathways in INp10 and *B. japonicus* and *B*. *platifrons* endosymbionts inferred from each genome and gene transcription, showing primary carbon metabolism (A) and respiratory systems (B). TPM values were ranked by top percentages among coding genes and illustrated with dot colors. Gray dots indicate the absence of genes. In cases where there are homologs and/or multiple genes involved in an enzyme, the most highly transcribed catalytic subunit gene was used for the level categorization. Note that transcription of INp10 and the endosymbionts is not strictly comparable. Abbreviations for pathways are as follows: ED, Entner-Doudoroff pathway; EMP, Embden-Meyerhof-Parnas pathway; MEP, methylerythritol 4-phosphate pathway; oxRuMP, oxidative ribulose monophosphate pathway; RGP, reductive glycine pathway; RuMP, ribulose monophosphate pathway. Abbreviations for substances are as follows: (C1 dissimilation and assimilation) H_4_MPT, tetrahydromethanopterin; MFR, methanofuran; H_4_F, tetrahydrofolate; Ru5P, ribulose 5-phosphate; H6P, 3-hexulose 6-phosphate; F6P, fructose 6-phosphate; GAP, glyceraldehyde 3-phosphate; E4P, erythrose 4-phosphate; X5P, xylulose 5-phosphate; S7P, sedoheptulose 7-phosphate; R5P, ribose 5-phosphate (ED and EMP); G6P, glucose 6-phosphate; 6PGL, 6-phosphogluconolactone; 6PG, 6-phosphogluconate, KD6PG, 2-keto-3-deoxy-6-phosphogluconate; DHA, dihydroxyacetone; DHAP, dihydroxyacetone phosphate; 1,3BPG, 1,3-bisphosphoglycerate; 3PG, 3-phosphoglycerate; 2PG, 2-phosphoglycerate; PEP, phosphoenolpyruvate; (others) G1P, glucose 1-phosphate; ADP-Glc, ADP-glucose; PRPP, 5-phosphoribosyl diphosphate; FDP, farnesyl diphosphate; 2,3-EDSQ, 2,3-epoxy-2,3-dihydrosqualene. Gene symbols (alphabetical order) are as follows in A: *aceE*, pyruvate dehydrogenase E1 component; *aceF*, pyruvate dehydrogenase E2 component; *cynT*, carbonic anhydrase; *CYP51*, sterol 14-demethylase; *dhaKL*, dihydroxyacetone kinase; *DHCR24*, delta24-sterol reductase; *eda*, 2-keto-3-deoxy-6-phosphogluconate aldolase; *edd*, 6-phosphogluconate dehydratase; *eno*, enolase; *fae*, 5,6,7,8-tetrahydromethanopterin hydro-lyase; *fchA*, methenyltetrahydrofolate cyclohydrolase; *FDFT1*, farnesyl-diphosphate farnesyltransferase; *fdh1AB*, formate dehydrogenase; *ffsA*, formylmethanofuran-tetrahydromethanopterin *N*-formyltransferase; *fhcABC*, formylmethanofuran dehydrogenase; *fhs*, formate-tetrahydrofolate ligase; *fsaA*, fructose-6-phosphate aldolase; *gapA*, glyceraldehyde-3-phosphate dehydrogenase; *gcvH*, glycine cleavage system H protein; *gcvP*, glycine dehydrogenase; *gcvT*, aminomethyltransferase; *glgA*, starch synthase; *glgB*, 1,4-alpha-glucan branching enzyme; *glgC*, glucose-1-phosphate adenylyltransferase; *glyA*, glycine hydroxymethyltransferase; *gnd*, 6-phosphogluconate dehydrogenase; *gpmI*, phosphoglycerate mutase; *hps*, 3-hexulose-6-phosphate synthase; *LSS*, lanosterol synthase; *mch*, methenyltetrahydromethanopterin cyclohydrolase; *mtdA*, methylenetetrahydrofolate-methylenetetrahydromethanopterin dehydrogenase; *mtdB*, methylene-tetrahydromethanopterin dehydrogenase; *oadABG*, oxaloacetate decarboxylase; *pck*, phosphoenolpyruvate carboxykinase; *pgi*, glucose-6-phosphate isomerase; *pgk*, phosphoglycerate kinase; *pgl*, 6-phosphogluconolactonase; *pgm*, phosphoglucomutase; *phi*, 6-phospho-3-hexuloisomerase; *pmoCAB*, particulate methane monooxygenase; *por*, pyruvate-ferredoxin/flavodoxin oxidoreductase; *pps*, pyruvate, water dikinase; *prsA*, ribose-phosphate pyrophosphokinase; *pycAB*, pyruvate carboxylase; *pyk*, pyruvate kinase; *rpe*, ribulose-phosphate 3-epimerase; *rpiA*, ribose-5-phosphate isomerase A; *shc*, squalene-hopene/tetraprenyl-beta-curcumene cyclase; *SQLE*, squalene monooxygenase; *tal*, transaldolase; *tkt*, transketolase; *tpiA*, triosephosphate isomerase; *xoxF*, lanthanide-dependent methanol dehydrogenase; and *zwf*, glucose-6-phosphate dehydrogenase. Gene symbols (alphabetical order) are as follows in B: *coxABC*, cytochrome *c* oxidase; *cydAB*, cytochrome *bd* ubiquinol oxidase; *narGHIJ*, nitrate reductase; *nirK*, nitrite reductase (NO-forming); *norBC*, nitric oxide reductase; *nqrABCDEF*, NADH:ubiquinone oxidoreductase; *petABC*, ubiquinol-cytochrome *c* reductase; and *sdhABCD*, succinate dehydrogenase.

Metabolic characterization of INp10 was performed by a transcriptome analysis of biofilms (methR-17d, methR-40d, and methR-88d). A summary of transcriptome sequencing results is presented in Table S3 in the supplemental material. Transcriptional profiles of selected metabolic pathways in INp10 were outlined along with profiles in the endosymbionts of *B. japonicus* and *B*. *platifrons* from Sagami Bay (Fig. 7; see Table S4 in the supplemental material showing detailed transcriptional data). Unfortunately, the RNA extracted from mussels collected from the Iheya North field had a low quality; thus, the high-quality RNA obtained from mussels collected in Sagami Bay was used in the analysis. The transcription levels were categorized by the ranking of transcripts per million (TPM) and are shown as follows: top >1%, extremely high; top >3% and >5%, very high; top >10% and >15%, high; top >30%, moderate; top >50%, low; and bottom 50%, very low (very high and high include two ranking categories to reduce the number of transcription levels and provide an overview of transcription) (see Table S5 in the supplemental material showing TPM values falling into each ranking category). This categorization is used for the following reasons: (i) a concern for the impacts of extremely high TPM values of *pmoCAB* encoding pMMO (accounting for 12% to 32% of the total TPM across all samples) on TPM of other genes; (ii) different patterns of gene distribution based on TPM between INp10 and endosymbionts (see Fig. S5 in the supplemental material); and (iii) concern about overestimating the differences in TPM between the samples that are not strictly comparable.

The properties of RNAs from INp10 are slightly varied among biofilms (Fig. S5). A statistical comparison of 53 ribosomal protein genes among biofilms demonstrated that their transcription rankings were significantly higher in the methR-17d than in the other two biofilms (*P < *0.001) (see Fig. S6 in the supplemental material). INp10 was likely more metabolically active in the methR-17d biofilm. Primary carbon metabolism profiles from transcription rankings did not appear to change markedly among biofilms.

### (i) Methane oxidation and carbon dissimilation/assimilation.

INp10 has basic pathways for carbon dissimilation/assimilation from methane (Table 2; Fig. 7A). The bacterium carries only a single *pmoCAB* cluster for methane oxidation. For subsequent methanol oxidation, only a single copy of *xoxF* encoding pyrroloquinoline quinone (PQQ)-dependent methanol dehydrogenase was identified. A putative solute-binding protein gene (*xoxJ*) adjacent to *xoxF* on the genome, a distantly carried cytochrome *c* gene (*xoxG*), and a PQQ synthesis gene cluster (*pqqABCDE*), all associated with *xoxF*, were identified. Formaldehyde can be oxidized to formate via parallel C_1_ transfer pathways, namely, tetrahydromethanopterin mediated and tetrahydrofolate (H_4_F) mediated. The oxidative ribulose monophosphate (RuMP) pathway is operational as another route for formaldehyde oxidation. Formaldehyde is fixed by the RuMP pathway as the serine pathway lacks hydroxypyruvate reductase.

Fructose 6-phosphate (F6P) is the branch-point in the RuMP pathway into the variants of glycolytic pathways, the Embden-Meyerhof-Parnas (EMP) and Entner-Doudoroff (ED) pathways (Fig. 7A). Notably, INp10 has neither 6-phosphofructokinase (both ATP and pyrophosphate dependent) nor fructose-1,6-bisphosphate aldolase-encoding genes. These enzymes produce glyceraldehyde 3-phosphate (GAP) from F6P via fructose 1,6-bisphosphate by consecutive reactions in the EMP pathway. Instead, INp10 has genes for F6P aldolase (*fsaA*) and dihydroxyacetone kinase (*dhaKL*) that compensate for the lack of canonical enzymes. The complete tricarboxylic acid cycle and anaplerotic enzymes to replenish oxaloacetate are encoded.

The reductive glycine pathway that produces glycine, serine, and pyruvate from formate, CO_2_, and ammonia may be partly functional in combination with the H_4_F-mediated C_1_ transfer pathway and the glycine cleavage/synthesis system (Fig. 7A). This pathway was first reported in genetically engineered Escherichia coli and later found in nonengineered bacteria (59, 60). INp10 may produce glycine and serine but not pyruvate through this pathway, as it lacks a serine deaminase gene.

Transcriptome analysis revealed extremely high to very high gene transcription for methane oxidation (*pmoCAB*), methanol oxidation (*xoxF* and *xoxG* but very low transcription of *xoxJ*), and the initial steps of formaldehyde dissimilation (*fae*) and assimilation (*hps*) (Fig. 7A; Table S4). Transcription of downstream enzymes involved in dissimilation reactions was much less than transcription of the abovementioned upstream reactions. Also, RuMP pathway enzymes generally maintained very high to high transcription, except for a few enzymes that act on branch-point substrates. One such substrate, ribose 5-phosphate (R5P), is converted to both ribulose 5-phosphate and 5-phosphoribosyl diphosphate; 5-phosphoribosyl diphosphate is essential for nucleotide synthesis. F6P is another branch-point substrate that enters glycolytic pathways. The ED pathway was likely dominant over the EMP pathway in GAP and pyruvate production. The genes for two key enzymes in the ED pathway *edd*, encoding 6-phosphogluconate dehydratase, and *eda*, encoding 2-keto-3-deoxy-6-phosphogluconate aldolase, were highly transcribed, but the transcription of *fsaA* and *pyk* encoding pyruvate kinase was low and very low, respectively.

### (ii) Characteristics associated with methane metabolism.

Hemerythrin is an oxygen-binding nonheme iron metalloprotein, and of the three hemerythrin gene homologs, the dominant gene was ranked within the top eight most-transcribed protein-coding genes (Fig. 7; Table S4), which likely indicates that INp10 was under oxygen-limited conditions within the biofilm structures. Whether hemerythrins carry oxygen to pMMO, terminal oxidases, or both is still debated (61, 62). Carbonic anhydrase (CA) was likely needed by INp10. The genome encodes five β-class CAs (*cynT*) of clades A, C, and D (63). Transcription varied among the clades; two clade D genes were dominant and one was extremely highly transcribed. The CAs of clade D were predicted to be cytoplasmic CAs, and the reason why INp10 appeared to require these CAs is currently unclear. Most of the CO_2_ produced by methane oxidation will diffuse out of the cells promptly under low partial pressure of CO_2_ (pCO_2_) conditions (63). However, diffused CO_2_ may have accumulated within biofilms, impeding rapid CO_2_ diffusion out of INp10 cells and possibly affecting CA expression.

Carbon can be stored as glycogen. The transcription of glycogen synthesis genes (*glgAB*) was low to very low, and yet glycogen-like intracellular granules were observed in methanotroph-like cells under TEM (Fig. 5B-4). Hopanoids and sterols are lipids that characterize bacterial methanotrophs and likely contribute to membrane stabilization and resistance to environmental stresses (64). These lipids are synthesized from isoprenoid precursors produced in the methylerythritol 4-phosphate pathway (Fig. 7A).

### (iii) Respiration and nitrogen metabolism.

Three terminal oxidases, namely, *caa_3_* and *b*(*o*/*a*)*_3_* types of cytochrome *c* oxidases and high-oxygen-affinity cytochrome *bd* ubiquinol oxidase, are encoded. The *caa_3_*-type oxidase appeared to be used more (Table S4). INp10 may be capable of partial denitrification from NO_3_^−^ to N_2_O with enzymes from genes encoding nitrate reductase (*narGHI*), NO-forming nitrite reductases (*nirK* and *nirS*), nitric oxide reductase (*norBC*), and a nitrate/nitrite antiporter (*narK*). One of two *nirK* homologs was very highly to highly transcribed. Notably, inverse transcriptional changes in biofilms were observed between *coxA* and *narG*, which are the catalytic subunits of *caa_3_*-type cytochrome *c* oxidase and nitrate reductase, respectively (Fig. 7B). A shift from aerobic to anaerobic respiration is likely to have occurred as the biofilm grew.

The transcript levels suggested that glutamine synthetase (*glnA*) contributed to nitrogen assimilation, but the assimilatory nitrate and nitrite reductases (*narB* and *nirBD*) were hardly involved (Table S4). One of two *hcp* homologs encoding hydroxylamine reductase was highly transcribed, suggesting the generation of toxic hydroxylamine from ammonia by pMMO. The genome also carries three truncated hemoglobins (trHbs), namely, a single gene of group I and two of group II. The group I trHb gene exhibited consistent and extremely high transcription and was ranked within the top 10 transcribed protein-coding genes. This high transcription level might have been a response to NO, as group I trHb is likely involved in resistance to nitrosative stress (65).

### (iv) Other functions advantageous to survival in hydrothermal systems.

INp10 has genes beneficial for organisms in environments with large local changes in conditions, e.g., oxygen, substances, and water current, such as hydrothermal vents (Table 2). The genome carries genes essential for flagellum assembly, which is consistent with the motility of methanotroph-like cells in culture. Homologous genes for components of the chemotaxis and aerotaxis systems were also identified. These systems function by sensing environmental and intracellular signals and controlling movement behavior (66). Ten homologs of methyl-accepting chemoreceptors and a single homolog of an aerotaxis receptor were found, and the aerotaxis homolog showed the highest transcription (Table S4). The presence of cellulose synthesis *bcsABZC* gene homologs is noteworthy. These genes are less common in methanotrophs. *bcsABZC* encodes BcsA and BcsB that form a catalytic complex regulated by cyclic di-GMP, an outer membrane porin BcsC, and an endoglucanase BcsZ; they are minimal but enough to construct a transmembrane cellulose synthase complex to produce and secrete properly assembled cellulose fibrils out of the cell (67). Gene transcription was low to very low. Cellulose, if produced, may be beneficial for biofilm construction (see Discussion). Meanwhile, some protein genes that are likely associated with adhesion and cell-to-cell interaction were extremely highly transcribed. They are homologs of a type IV pilus assembly protein, PilA, a trimeric autotransporter adhesin, a C-type lectin domain protein, and three lipoproteins (Table S4). (68). They are predicted to locate on the cell surface or in the outer membrane. An extremely high transcription level was also found for some genes encoding proteins with the PEP-CTERM protein-sorting domain associated with exopolysaccharide production (69).

Other genes associated with survival and environmental stress response were also identified. The type VI secretion system, a cell-to-cell contact-dependent offensive system that delivers effector proteins into targets in one step (70), appeared activated in biofilms. An extremely high transcription level was observed for a homologous gene of an Hcp protein involved in the building of tubular structures extending outward from the cells (Table S4). Intriguingly, INp10 may have the potential to produce enediynes, known as antitumor antibiotics, as harboring homologs of the *sgcE* cluster (*E3*, *E4*, *E5*, *E*, and *E10*) commonly found for all enediyne polyketide synthases (71). The transcription levels were, however, low to very low. The genes involved in many toxin-antitoxin (TA) systems were identified in the genomes of *Methyloprofundus* spp., including INp10, using the TADB 2.0 database (72) (Table 2). This search showed mutually distinct patterns of TA systems. At least 57 TA systems were predicted and 39 were not orthologous to others. Activation of several TA systems was suggested, including the YoeB-YefM, MazEF, and BrnTA families involved in biofilm formation, programmed cell death, and control of bacteriostasis, respectively (73).

### Summary of the genetic characteristics of *B. japonicus* and *B*. *platifrons* endosymbionts.

The integrated genomic and transcriptomic analysis of the endosymbionts of *B. japonicus* and *B*. *platifrons* suggested that they have essentially the same basic pathways for methane oxidation and carbon fixation as INp10. Methane metabolism appeared active *in situ* based on the transcriptome analysis (Fig. 7; see Table S4 showing transcriptional data of symbiont orthologs of INp10 genes). The genomes showed a difference in their glycolytic pathways. The *B. japonicus* endosymbiont has only the EMP pathway, whereas the *B*. *platifrons* endosymbiont has both EMP and ED pathways as in INp10. Notably, their EMP pathway is also an unusual one that, like in INp10, does not include a route via fructose-1,6-bisphosphate. Activation of the EMP pathway was inferred in both endosymbionts from the high transcription levels of *fsaA* and *pyk*. Both endosymbionts lack genes encoding anaplerotic enzymes to produce oxaloacetate, although they have *maeA* encoding malate dehydrogenase (oxaloacetate decarboxylating) to produce malate from pyruvate. This gene is absent in INp10. The endosymbionts will also store carbon as glycogen, and glycogen synthesis appeared very active.

The characteristics supposedly associated with a symbiotic lifestyle are absence of flagella, pili, chemotaxis, aerotaxis, and type VI secretion systems (Table 2); these systems are supposed to be beneficial for a free-living lifestyle. Furthermore, the *B. japonicus* endosymbiont might be less adapted to hypoxia due to the absence of hemerythrin, and the transcription level was low for the sole hemerythrin gene in the *B*. *platifrons* endosymbiont. Both endosymbionts lack genes for high-oxygen-affinity cytochrome *bd* ubiquinol oxidase. The endosymbionts appear less prepared for hypoxia than free-living species, probably due to a sufficient oxygen supply from their hosts. However, they have genes for partial denitrification (from NO_3_^−^ to NO) and highly transcribed *narG*. The endosymbionts will assimilate nitrogen mainly via GlnA. Their group I trHb genes were extremely highly to very highly transcribed, as observed for INp10, possibly in response to nitrosative stress.

## DISCUSSION

### Properties of ISCS communities.

This study used ISCSs with the expectation of obtaining active microorganisms inhabiting the deep-sea hydrothermal field. The proportion of *Methylococcales* in ISCS communities was unexpectedly high. To the best of our knowledge, this finding has not been reported previously. An exceptional result was recorded from ISCS-4, one of the two ISCSs that showed signs of heat exposure. A high proportion of *Campylobacteria* was detected as observed in previous reports (74, 75) (Fig. 2A). Local differences might exist in community composition within a colony, especially for *S. crosnieri* colonies where the environmental conditions greatly fluctuate in diffusing hydrothermal flows, as inferred from the discrepancy between the signs of heat exposure on the ISCSs and the low-temperature records.

The ISCS community will not necessarily directly mirror indigenous community composition in its surroundings, although we could not verify this assumption due to the limitations of the field survey. SEM and FISH analyses identified biofilms and clusters of similarly shaped cells from the ISCSs, suggesting that communities are likely composed mainly of microorganisms grown in ISCSs for 2 months with few proportions of simply attached cells. The ISCS deployment time can also affect community composition. Gulmann et al. (76) reported successive composition changes and increasing diversity in biofilm communities on basalt blocks over a 9-month period at diffuse-flow vents. In this context, ISCS communities are a snapshot of changing community development.

Nakamura and Takai (77) presented a theoretical calculation proving that methane and sulfur oxidation can be two major energy metabolisms and can generate almost equal metabolic energy yield at moderate-to-low temperatures in hydrothermal fluid-seawater mixing zones in this field. Thus, the high proportion of *Methylococcales* seems consistent with this energetic prediction. However, the MOB population may not be proportional to theoretical energy yield because they frequently spill energy-producing methanol and/or other metabolites from cells (78–80). This trait leads to cross-feeding between MOB and other microbes, including methylotrophs, and lowered proportions of MOB. Such cross-feeding surely occurred in the enrichment culture in this study and has also been reported in natural habitats (81, 82).

Identification of 161 species-like groups of *Methyloprofundus* is notable despite the above factors affecting the community structure. The poor consistency between 16S rRNA gene and *pmoA* amplicon analyses may reflect differences in species-level resolution and may also be caused by primer bias. The proposed 4% cutoff value for *pmoA* for species delineation (24) seems acceptable at this time for *Methyloprofundus* spp., as the smallest *pmoA* sequence difference is 4.5% between the comparisons of four independent species for the about 500-bp-long region sequenced conventionally. The diversity of species-like group populations from ISCSs is likely higher at colonies of bathymodiolin mussels than at *S. crosnieri* colonies, although the number of samples is too small to support conclusions. Hydrothermal fluid input is generally more pronounced at *S. crosnieri* colonies, and fluctuating conditions, such as temperature, pH, hydrogen sulfide, and metals, may put selective pressure on members of *Methyloprofundus* and reduce diversity.

Much remains unclear about the free-living pre-endosymbionts of bathymodiolin mussels, which we define here as endosymbionts living outside the cells of mussels. Proportions of free-living pre-endosymbionts were low, if any, in ISCS communities. Nevertheless, even if many relatives of endosymbionts exist near mussels, the mollusks still identify their endosymbiotic partners. The mechanisms for partner recognition are not fully understood.

### INp10 may represent a dominant species within the *Methyloprofundus* population.

We successfully enriched INp10, a new species of *Methyloprofundus*, which is likely a relatively dominant species in the *in situ Methyloprofundus* population. Surprisingly, we independently enriched two more methanotrophs that shared identical 16S rRNA gene sequences with INp10. The plausible reasons for these repeated enrichments are (i) the high abundance in this field, (ii) the overlap of preferred growth conditions and cultivation conditions, and (iii) their fitness for survival under unfavorable conditions experienced from sampling to cultivation. In association with the third reason, INp10 is inferred from its genome to have characteristics such as tolerance to hypoxia, environmental sensing, motility, adhesion, biofilm formation, and attack devices. These characteristics may derive from systems, including partial denitrification, aerotaxis, chemotaxis, flagella, pili, cellulose synthesis, type VI secretion system, and enediyne synthesis. These systems are not necessarily unusual; however, their combination may allow INp10 to better compete for habitat with other deep-sea species. Interestingly, *M. sedimenti* WF1^T^, isolated from deep-sea sediment, appears to lack these systems, except for partial denitrification and pili (54). The sediment environment will be more stable than the hydrothermal environment. Hence, these systems lack importance for living in sediment.

The predicted synthesis of enediyne and cellulose is notable as they are unusual in methanotrophs. Enediynes are among the most promising antitumor antibiotics, with unique chemical structures and extraordinary cytotoxicity (71). Enediyne synthase genes are found mostly in the phylum *Actinobacteria*; however, a database search found homologs of the conserved marker genes in two *Methylotuvimicrobium* species (71). The presence of other related genes is unclear, and the actual synthesis of functional enediynes is not demonstrated; if such synthesis is possible, it would be advantageous in competition with other species.

Bacterial cellulose, a chemically stable polymer, can be a component of biofilms. *Proteobacteria* include many cellulose producers. Studies in plant- and animal-associated bacteria suggest that cellulose contributes to stable adhesion and colonization, biofilm maturation, and protection against environmental stress (67, 83). The deletion or mutation of *bcs* caused impairment in adhesion and biofilm formation in some species (84, 85). The database search for *bcsABZC* homologs identified genes in some gammaproteobacterial methanotrophs, including *Methylotuvimicrobium*, *Methylomarinum*, and *Methylomarinovum* species, and uncultured deep-sea and freshwater species. The *bcs* transcription in INp10 was low. We detected a small amount of cellulose (approximately 0.6%, vol/vol) in alkali-washed mature biofilms (data not shown), but this rough detection needs to be further confirmed via more careful measurement. If INp10 produces cellulose, it might be used in the initial adhesion step for a solid foundation. Given that INp10 and its close relative methanotroph were cultivated from mussel gills, they might colonize the gill surface, possibly mimicking the use of cellulose as a support, as observed in other animal-associated cellulose producers (67, 83). Unfortunately, we have not verified such colonization. INp10 and the gill endosymbionts are too similar to be distinguished by FISH.

Two types of methanol dehydrogenases are known, namely, calcium-dependent MxaFI and lanthanide-dependent XoxF. Most proteobacterial methanotrophs carry genes for both enzymes, namely, *mxaFI* and *xoxF*. In *Methyloprofundus*, *M. sedimenti* WF1^T^ and Bathymodiolus puteoserpentis endosymbiont (NZ_FQTQ00000000) follow suit, carrying both genes. However, only *xoxF*, which belongs to clade 5 (Xox5) (86), is present in INp10 and the endosymbionts of *B. japonicus*, *B*. *platifrons*, and Bathymodiolus azoricus (87). The presence of *xoxF* alone was also reported in the MMG2 clade (18, 88). XoxF receives attention as a rare example of an enzyme containing a lanthanide ion in the active site (89, 90). Light lanthanides generally induce *xoxF* transcription, and verrucomicrobial methanotrophs carrying only *xoxF* require nanomolar levels of lanthanide supplement for growth (90, 91). We have consistently cultivated INp10 without lanthanide supplement; thus, XoxF5 in INp10 may utilize contamination levels of lanthanides.

Studies on lanthanide uptake suggest the involvement of a TonB-dependent outer membrane receptor to transport chelated lanthanides (86, 92, 93); LanA is one such receptor identified in Methylotuvimicrobium buryatense 5GB1C (94). Although no homolog of LanA, INp10 has eight gene homologs of the TonB-dependent receptor for siderophores and two were highly transcribed in the methR-17 biofilm (Table S4). MxaY and MxaB are assumed to be components of the lanthanide-dependent system regulating MxaFI and XoxF expressions (95–97). INp10 has genes for a sensor histidine kinase (methR_P0273) and a response regulator (methR_P0261), sharing 58% and 69% amino acid identities to MxaY and MxaB, respectively, of *M. buryatense* 5GB1C. Nevertheless, the lanthanide-dependent regulation in INp10 carrying only *xoxF* may be simpler than what we know.

The lanthanide availability in the habitat of INp10 is uncertain. A deep-sea hydrothermal field in the Southern Okinawa Trough shows nanomolar levels of lanthanides in hydrothermal fluids, but mixing water near vents showed only picomolar levels due to a rapid decrease in solubility (98). Uptake of picomolar levels of light lanthanides by probable methanotrophs was observed in the water column in the Gulf of Mexico during the oil/gas spill accident (99, 100). MOB living at such low lanthanide concentrations may have developed high-affinity lanthanide uptake systems.

The unusual glycolytic EMP pathway exists in the *Methyloprofundus* species that we evaluated. This pathway employs FsaA, an F6P aldolase first found in Escherichia coli in 2001 (101), and DhaKL. The *B. japonicus* endosymbiont lacks the ED pathway, and both endosymbionts exhibit elevated gene transcription for FsaA and DhaKL. Hence, the EMP pathway will certainly function for the endosymbionts. Because the RuMP pathway substrates are used for *de novo* synthesis of, for example, saccharides and nucleotides, the FsaA reaction in the gluconeogenic direction to produce F6P is also important.

The EMP pathway generates more ATP than the ED pathway. Meanwhile, the advantage of the ED pathway in terms of glycolytic efficiency has been presented by a thermodynamic and kinetic comparison (102). Probably the primary role of glycolytic pathways for methanotrophs is not ATP production but instead the production of pyruvate and intermediates. Hence, in principle, the ED pathway seems favorable to obligate methanotrophs, and accordingly, INp10 likely favors the use of the ED pathway. However, the EMP pathway appeared dominant over the ED pathway in the *B*. *platifrons* endosymbiont, similar to that previously reported in the *Methylotuvimicrobium* and *Methylomonas* species (78, 103, 104). The physiological status of INp10 grown under nonoptimized conditions may be different from pure-cultured fast-grown cells. Such altered growth conditions might affect the balance between pathways.

### Implications for the global distribution of free-living *Methyloprofundus* spp.

The distribution of *Methyloprofundus* spp. is not well understood, probably because its members are, in most cases, minor components of their communities. *Methyloprofundus*-like sequences are typically detected from deep-sea environments. However, their distribution is not likely confined to the deep sea. Similar sequences have been detected from shallow seawater (1.5 and 7 m deep) at subzero temperatures in the Arctic Ocean (105) and from shallow sediment (21 m deep) off Santa Barbara, CA (106), where the seawater temperature is governed by the cold California current. Notable detection is also reported in the water column at a depth of 32 m in the South China Sea, where the water temperature is approximately 27°C, which is above the upper limit for the growth (26°C) of *M. sedimenti* WF1^T^ (17, 107). Despite these reports, the limited number of detections in shallow waters indicates that low temperature is likely a key factor for most *Methyloprofundus* species. Some may, however, be adapted to moderate temperatures. A temperature difference of only a few degrees may define *Methyloprofundus* species composition, partly explaining differences in ISCS communities.

Previous detection of *Methyloprofundus*-like sequences in oceanic oxygen minimum zones (9, 42) may reflect the adaptation of *Methyloprofundus* spp. to low oxygen concentrations. The presence of genes for partial denitrification from nitrate in the four *Methyloprofundus* species compared and the *B. puteoserpentis* endosymbiont (NZ_FQTQ00000000) is consistent with this hypothesis. In addition, partial and possibly disrupted *nar* genes are present in the endosymbiont of *Bathymodiolus* sp. (FNVW00000000) from the Mid-Atlantic Ridge. The nitrate-reducing ability may be or may have been shared by many *Methyloprofundus* species.

The concentration of nitrate in many natural environments is several times higher than that of nitrite (108, 109); thus, nitrate-reducing ability is advantageous for survival under hypoxic conditions. A search of databases and published reports showed that gammaproteobacterial methanotrophs with intact dissimilatory nitrate reductase genes (*narGHI* or *napAB*) are distributed in five genera (*Methylomonas*, *Methylobacter*, *Methylosarcina*, *Crenothrix*, and *Methyloprofundus*) and uncultured species, such as *Methylothermaceae* (110), the MMG2 clade (88), and the OPU3/deep-sea 3 clade (9). Methylomonas denitrificans FJG1 can couple aerobic methane oxidation to nitrate reduction at low O_2_ levels (111). However, nitrate reductase genes are unlikely to be common in each of these genera.

Overall, the *Methyloprofundus* clade is relatively common in cold marine ecosystems, including hypoxic environments, but is overlooked due to its low abundance. If its ubiquity is real, it may explain why cosmopolitan bathymodiolin mussels consistently select *Methyloprofundus* spp. as methanotrophic endosymbionts. *Methyloprofundus* spp. may always be present in new settlements of bathymodiolin mussels, and both species may prefer similar habitats.

In conclusion, this study focused on unidentified free-living *Methyloprofundus* spp. in the Iheya North deep-sea hydrothermal field. The results indicated the presence of many free-living species at the colonies of the bathymodiolin mussels and galatheoid crab. Furthermore, the possible effects of hydrothermal input on species composition and diversity in the *Methyloprofundus* population were presented. The behavior of the free-living pre-endosymbionts of bathymodiolin mussels remains unclear. Genomic and transcriptomic characterization of cultivated *Methyloprofundus* sp. INp10 showed its adaptation for survival under field conditions. At present, available data on comparable free-living species are too small to elucidate the relationships among phylogeny, habitat preference, and genomic and physiological traits. A more elaborate combination of molecular analysis and environmental monitoring will provide a comprehensive understanding of the *Methyloprofundus* clade. Finally, the high proportion of the poorly understood MMG2 clade to which *S. crosnieri* epibionts belong (47) in the MOB population from ISCS suggests that this hydrothermal field is promising for further study of two fascinating marine-specific methanotrophic clades that harbor chemosynthetic animal symbionts.

## MATERIALS AND METHODS

### Sample collection and experiments.

All samples and data from the deep sea were collected using the remotely operated vehicle (ROV) *Hyper-Dolphin* during the cruises NT09-17 (October 2009), NT10-E01 (August 2010), NT13-07 (April 2013), NT13-22 (November 2013), NT14-05 (April 2014), and NT15-13 (July 2015) operated by R/V *Natsushima* and KY14-01 (January 2014) operated by R/V *Kaiyo*. These cruises were organized by JAMSTEC. The samples and experiments performed in this study are summarized in Table S6.

### *In situ* colonization systems.

ISCSs were deployed for 2 months in the Original site in the Iheya North deep-sea hydrothermal field, the mid-Okinawa Trough, East China Sea (Fig. 1A). The ISCSs consisted of porous ceramic particles for fish tank filters (SUDO, Nagoya, Japan) (Fig. 1B-1), a stainless steel basket, and a sinker to settle into the sea bottom (Fig. 1B-2). Before the ISCSs were assembled, the ceramic particles were heated at 400°C for 3 h to remove organic matter. The ISCSs (ISCS-1 to ISCS-4) were deployed at four animal colonies during the NT13-22 cruise in November 2013 and recovered during the KY14-01 cruise in January 2014. The ceramic particles in the ISCSs were immediately subsampled and treated on board as follows: stored at −80°C for molecular analyses; fixed with 4% paraformaldehyde in filter-sterilized Rei-Sea marine seawater (IWAKI, Tokyo, Japan) for 16 h at 4°C, rinsed with phosphate-buffered saline (PBS) twice, and stored in 50% ethanol in PBS at −80°C for molecular and microscopic analyses; and stored at 4°C under N_2_ atmosphere for cultivation.

### Bathymodiolin mussels.

An individual of *B. japonicus* as an inoculum for the cultivation of methanotrophs was collected at a depth of 986 m from the foot of the NBC in the Original site in the Iheya North deep-sea hydrothermal field during the NT09-17 cruise (Fig. 1A). The mussels were kept alive without methane supply before cultivation was started. Another individual of *B. japonicus* for the cultivation of methanotrophs was collected about 100 m southeast of the NBC vent at a depth of 1,023 m during the NT10-E01 cruise. The gill tissue was immediately dissected on board and stored in a vial under N_2_ at 4°C before cultivation was started.

Individuals of *B. japonicus* and *B*. *platifrons* for genomic and transcriptomic analyses of their endosymbionts were collected from the off Hatsushima seep in Sagami Bay, Japan (35°01′N, 139°13′E; 857 m and 957 m depth) (Fig. 1A) during the NT13-07 and NT14-05 cruises. *In situ* RNA stabilization for the endosymbionts at the habitat was performed during the NT14-05 cruise as described previously (44, 112). Briefly, mussel individuals were collected in a sample container using the ROV manipulator. The container was filled beforehand with a stabilization solution containing nearly saturated sulfate salt (25 mM sodium citrate [pH 5.2], 10 mM EDTA, and 700 g/L ammonium sulfate) to protect RNA. In this ROV manipulation, shells of mussels were gently broken by the manipulator to allow the stabilization solution to penetrate tissues of mussels and then transferred into the container, and the container was replenished with the stabilization solution. After recovery on board the ship, the samples were washed three times using the chilled stabilization solution to reduce contamination. The dissected gill tissues were stored in RNAlater (Ambion) at 4°C overnight and −80°C afterward.

### Measurement of environmental conditions.

The environmental characterization of the ISCS-deployed sites was carried out upon the ISCS recovery during the KY14-01 cruise, except for methane measurement that was performed during the NT15-13 cruise at approximately the same sites.

Methane, H_2_S, and H_2_ were measured using *in situ* sensors. A methane sensor (METS; Franatech, Lüneburg, Germany) and a sensor system fitted with electrodes for H_2_S and H_2_ (Unisense, Aarhus, Denmark) were combined with a water sampling system equipped with the Hachiren water sampler as described previously (113). Dissolved oxygen (DO) was measured using an *in situ* phosphorescence DO sensor (RINKO I, ARO-USB; JFE Advantech, Hyogo, Japan). Temperatures were measured using the above DO sensor or a platinum-resistance electronic temperature sensor (NiGK Corp., Saitama, Japan) installed in the above water sampling system. The pH of the seawater collected by the Hachiren sampler was determined on board.

### Cultivation of methanotrophs using continuous flowthrough systems.

The gill tissue of a living individual of *B. japonicus* collected during the NT09-17 cruise was dissected 5 days after the sampling. The gill was homogenized in an ice-cooled mortar and spread on two pieces of polyethylene nonwoven fabric (NWF; 1.5 by 5 cm). Each piece was set in a glass column (3 cm in diameter, 9.5 cm in length; Bio column KF-30; TGK, Tokyo, Japan) connected to a continuous flowthrough cultivation system (Fig. 5A). Five liters of filter-sterilized medium was prepared in a glass bottle, and the gas phase of the bottle was filled with 100% methane at least 1 day before use. The medium was supplied periodically; a 1-min supply at 3 mL/min followed by a 19-min pause was repeated using an electronic on-off timer that controlled a peristaltic pump with Viton tubing (Masterflex L/S 7550-50; Cole-Parmer, Vernon Hills, IL, USA). A gas-tight aluminum bag filled with a mixed gas of N_2_, CH_4_, and O_2_ (83.8:15:1.2) was connected to the medium bottle. The cultivation system was continuously operated at 10°C.

The initial cultivation medium was prepared at pH 6.7 and 7.5, with 10 mg NaNO_3_, 5 mg Na_2_HPO_4_, 2 mg KH_2_PO_4_, 0.025 mg CuSO_4_·5H_2_O, and 2-mL DSMZ 141 trace element solution per L of Rei-Sea marine seawater (IWAKI). After methanotrophic growth was observed at pH 7.5, several minor changes were made in cultivation conditions and then fixed before further experiments were conducted as follows. The medium (pH 7.5) was prepared with 0.1 g NaHCO_3_, 29 mg NH_4_NO_3_, 5 mg Na_2_HPO_4_, 2 mg KH_2_PO_4_, 0.025 mg CuSO_4_·5H_2_O, and 1.6-mL trace element solution per L of Rei-Sea marine seawater and supplied at 20 mL/min for 1 min followed by a 1-h pause. Dissolved methane and DO concentrations in the medium were measured on a separately assembled cell-free cultivation system. DO was measured by sealing a probe of the DO meter (HQ40d; Hach, Loveland, CO, USA) in the column through an extra outlet. Dissolved methane was extracted from 0.5 mL of the medium in a vacuumed vial and measured using a gas chromatograph (GC-4000; GL Science, Tokyo, Japan) with a molecular sieve 5A column and a helium ionization detector (VICI Valco Instruments, Houston, TX, USA). The measurement was conducted once a day during the 10 days of operation.

The cell morphology of the culture was routinely observed with a BX51 microscope (Olympus, Tokyo, Japan). The presence of methanotrophs was examined by *pmoA* amplification as described previously (114). If the medium flow decreased because of clogged fritted glass discs set in the inlet and outlet of the column by overgrown biofilms, which usually occurred at intervals of about a year, the enrichment culture was subcultured by transferring an old piece of NWF to a new column with a new piece of NWF. This subculturing was also performed before each experiment, and the growth period after the subculturing is shown in each experiment.

The biofilm growth rate was calculated by measuring the increase in protein content as follows. Pieces of a cut glass slide (15 by 25 mm) were placed in the column as scaffolds of biofilm and sampled every few days within 2 weeks. They were heated in 5% sodium dodecyl sulfate (SDS) solution for 10 min to lyse all attached cells. The cell lysate was diluted with distilled water and subjected to a protein assay using a bicinchoninic acid (BCA) protein assay kit (Thermo Fisher Scientific, Waltham, MA, USA). Four independent experiments were performed, and an average doubling time was calculated. The biofilm samples were subjected to electron microscopy, FISH, and DNA and RNA analyses as described later.

The other two independent cultivation experiments were performed at 5°C in similar systems, as described above, using the gill tissue of *B. japonicus* 4 days after sampling during the NT10-E01 cruise and a mixed sample of ISCS-2 or ISCS-3.

### Metabolite measurement in the culture supernatant.

The concentrations of methanol, formate, and acetate in the culture supernatant were measured. A 10 mL of culture was collected from the column every hour, three times in total, at the time just before the peristaltic pump worked. Each sample was immediately filtered with a 0.22-μm Millex-GP filter (Merck, Darmstadt, Germany) that was pre-washed three times with distilled water to remove any solutes from the filter. Methanol was measured by using an Agilent 6890N gas chromatograph coupled with an Agilent 7694 headspace sampler (Agilent, Santa Clara, CA, USA). Formate and acetate were derivatized with 2-nitrophenylhydrazine using the fatty acid analysis kit (YMC, Kyoto, Japan) and measured by a high-performance liquid chromatography (HPLC) system with a CBM-20A system controller and an SPD-10A UV/VIS detector (Shimadzu, Kyoto, Japan) on a Nova-Pak C_18_ column (3.9 by 150 mm; Waters, Milford, MA, USA) at 45°C. The eluents were acetonitrile containing 0.1% trifluoroacetic acid (A) and distilled water (B), and the following gradient elution program was run: 1.0 mL/min with 7.5% A in 45 min, 100% A in 10 min, and 7.5% A in 10 min. The medium was used as a negative control. The independent sampling and measurement were repeated three times.

### Electron microscopy.

For SEM observations of the ISCS samples, the ceramic particles fixed on board were further fixed with 2.5% glutaraldehyde and prepared as described previously (115), with a modified drying method in which the samples were freeze-dried using *t*-butyl alcohol. Observations were performed using a field-emission SEM (FE-SEM; JSM-6700F; JEOL, Tokyo, Japan) at 5 kV. To determine the thickness of biofilms on the particle surface, cross-sectional milling of biofilms was performed using an SEM equipped with a focused ion beam (FIB-SEM; Helios G4 UX DualBeam; FEI/Thermo Fisher Scientific) with a gallium ion beam of 30 kV and a processing current of 1.2 to 9.1 nA. The biofilm thickness was measured by SEM at an acceleration voltage of 1 kV, and the measurements of thickness were corrected for angle.

The cultivated biofilm sample was fixed with 2.5% glutaraldehyde and prepared as described previously (115). The sample was observed by FE-SEM as described above. The sample for TEM observation was prepared as described previously (116) with some modifications. In brief, the biofilm was frozen rapidly and freeze-substituted in acetone containing 2% osmium tetroxide at −80°C for 2 to 6 days. The biofilm was further subjected to *en bloc* staining with 1% uranyl acetate in methanol to increase contrast and embedded in epoxy resin. Ultrathin sections were observed by using a Tecnai 20 TEM (FEI/Thermo Fisher Scientific) at 120 kV.

### Fluorescence *in situ* hybridization.

Whole-cell FISH was performed on the ISCS samples, except for ISCS-2, which was not properly fixed on board. The fixed ceramic particles in the ISCSs were weakly sonicated (40 kHz, 5 s, three times) in 50% ethanol in PBS with cooling. The fine ceramic pieces peeled off from the particle surface were collected in 1.5-mL tubes and washed in PBS, and subsequent processes were performed in the tubes. For whole-cell FISH on the cultivated biofilm, the sample was harvested by centrifugation at 5,000 × *g* for 5 min at 4°C, fixed with 4% paraformaldehyde in PBS (4°C, 16 h), rinsed in PBS three times, and then stored in 50% ethanol in PBS at −30°C. For a negative-control sample, exponentially grown cells of Methylobacter marinus MR1, a marine methanotroph isolate, were prepared in the same manner as the biofilm. Samples of the biofilm and MR1 were dropped onto prewarmed (46°C) glass slides; left until dry (15 min); washed with 50%, 80%, and 100% ethanol; and then air-dried before hybridization.

A new probe, Mp731 (5′-CAGTTTTAGTCCAGGAAGTCGC-3′), was designed for 16S rRNA of the *Methyloprofundus* clade using the ARB software package (117) and the SILVA database (118). Mp731 covered 93% of the available *Methyloprofundus* sequences (including sequences of strain INp10 and *B. japonicus* and *B*. *platifrons* endosymbionts) and also 100% of *Methyloprofundus* ASVs_-16S_ obtained from the ISCSs in this study. In the other two dominant methanotrophic clades detected in this study, the MMG2 clade had at least one-base mismatch but mostly two-base mismatches or more, whereas the pLW-20 clade had two-base mismatches or more with Mp731 in the sequences of the SILVA database and ASVs_-16S_. The negative control *M. marinus* MR1 carried a two-base mismatch, which was found not to hybridize with MP731 but hybridized with Eub338. The probe specificity is summarized in Table S7 in the supplemental material. The Eub338 (119) and NonEub (reverse complementary of Eub338) (120) probes were used as positive- and negative-control probes for bacterial 16S rRNA, respectively (see the probe sequences in Table S8 in the supplemental material), and NonEub was found not to hybridize any samples.

Hybridization was performed at 46°C in a solution containing 20% formamide, 0.9 M NaCl, 20 mM Tris-HCl (pH 7.5), 5 mM EDTA, 0.01% SDS, and 0.5 μM of each probe. The MP731 probe was labeled with Alexa Fluor 488, whereas the Eub338 and NonEub probes were labeled with Alexa Fluor 555. After hybridization, the samples were washed twice in a solution containing 0.225 M NaCl, 20 mM Tris-HCl (pH 7.5), 5 mM EDTA, and 0.01% SDS at 48°C for 20 min to remove excess probes. The samples were mounted in Vectashield with 4′,6-diamidino-2-phenylindole (DAPI; Vector Laboratories, Burlingame, CA, USA). The ceramic pieces hybridized in 1.5-mL tubes were spread onto glass slides for microscopy. FISH images were captured using an A1RMP confocal scanning system (Nikon Solutions, Tokyo, Japan) with some *z*-axis scanning, and the maximum intensity projections were acquired using NIS-Elements software (Nikon Solutions).

### DNA and RNA preparations.

To extract DNA from the ISCSs, five ceramic particles (about 4 g) from each ISCS were ball-milled in an ice-precooled 50-mL stainless steel jar with a 25-mm stainless steel ball for 5 s at 30 Hz using a Mixer Mill (MM301; Retsch, Haan, Germany). For the *pmoA* analysis, two more replicates of ball-milled samples were prepared. DNA was extracted from 0.7 to 1 g each of the ball-milled samples using a DNeasy PowerSoil kit or a DNeasy PowerSoil Pro kit (Qiagen, Hilden, Germany) according to the manufacturer’s instructions. Upon extraction, each sample was divided into 3 to 5 bead tubes and treated, and the extracted DNA was combined. A negative-control extraction was performed with the ceramic particles not utilized for ISCSs.

In the analyses of the enrichment culture, the biofilms cultivated for 17, 40, and 88 days (methR-17, methR-40, and methR-88) on NWF (1.5 by 6 cm per each sample) after subculturing were collected for DNA and RNA at the same time, and the samples for RNA were promptly immersed in RNAprotect bacteria reagent (Qiagen). All biofilm samples were stored at −80°C until used. DNA and RNA from the biofilms were extracted using PowerBiofilm DNA and PowerBiofilm RNA isolation kits (Mo Bio Laboratories, Carlsbad, CA, USA), respectively, according to the manufacturer’s instructions. Upon extraction, each NWF was cut into 4 pieces, and nucleic acids extracted from each piece were combined.

Genomic DNA of the endosymbionts was extracted from the gill tissue of one individual each of *B. japonicus* and *B*. *platifrons* using a DNeasy blood and tissue kit (Qiagen). Prior to DNA extraction, the endosymbiont cells in the gill tissues were enriched using the method described previously (121) to reduce host DNA contamination. The total RNA of the endosymbionts was extracted from the *in situ* fixed gill tissue of one individual each of *B. japonicus* and *B*. *platifrons* using the NucleoSpin RNA kit (Macherey-Nagel, Düren, Germany).

The extracted DNA and RNA were quantified using Qubit assay kits on a 1.0 fluorometer (Invitrogen, Carlsbad, CA, USA). The RNA quality was checked with an Agilent RNA 6000 Pico kit on a 2100 bioanalyzer (Agilent). All samples were kept at −80°C until used.

### 16S rRNA gene amplicon sequencing.

The variable regions of 16S rRNA genes were amplified from DNA from the ISCSs and the cultivated biofilms, with primers 515F and 806R (122) for the ISCSs and primers U530F and U907R (123) for the biofilms. The forward and reverse primers were added with an Illumina adaptor sequence and an Illumina multiplexing PCR primer 2.0 sequence at the 5′ ends, respectively. Amplification was performed as described previously (124) using LA *Taq* polymerase and 2× GC buffer I (TaKaRa Bio, Shiga, Japan). The first PCR conditions were as follows: 96°C for 1 min; 25 cycles of 96°C for 20 s, 50°C (515F/806R) or 52°C (U530F/U907R) for 45 s, and 72°C for 1 min; and 72°C for 7 min. The previously reported primer and adaptor information is shown in Table S8.

The PCR products were checked by agarose gel electrophoresis and purified using the ExoSAP-IT PCR product cleanup reagent (Affymetrix, Santa Clara, CA, USA). The second PCR was performed to add multiplexing indices and Illumina sequencing adapters using *Ex Taq* polymerase (TaKaRa Bio). The adapter-attached PCR products were purified with Agencourt AMPure XP (Beckman Coulter, Brea, CA, USA) and quantified using the Quant-iT double-stranded DNA (dsDNA) high-sensitivity assay kit (Life Technologies, Carlsbad, CA, USA). The quality and concentration of PCR products were checked using an Agilent 2100 bioanalyzer and real-time PCR with a library quantification kit (Kapa Biosystems, Wilmington, MA, USA). Sequencing was performed on the MiSeq system (Illumina, San Diego, CA, USA) using MiSeq reagent kit v3, as recommended by the manufacturer.

### *pmoA* gene amplicon sequencing.

In the *pmoA* analysis targeting the *Methyloprofundus* clade, new primers were designed. Six *pmoA* sequences from the *Methyloprofundus* clade were aligned with 33 sequences from isolates of representative genera of the order *Methylococcales*. The better primer set containing MPFpmo-F1 (5′-ACTGTAGCGCCAATCGTATC-3′), MPFpmo-F2 (5′-ACTGTAGCGCCGATCGTTTC-3′), and MPFpmo-R (5′-ACTGGAGCAACRTCTTTACC-3′), which amplified 461-bp products, including primers, was selected by using Primer 3 software. The MPFpmo-R primer site was at nearly the same position as that of the common mb661 primer (125). Subsequent procedures were performed basically in the same manner as those in the 16S rRNA gene analysis. The first PCR on DNA from the ISCSs was performed using the above primers with the Illumina sequences (0.2 μM each) with the following PCR conditions: 96°C for 1 min; 30 cycles of 96°C for 20 s, 55°C for 45 s, and 72°C for 30 s; and 72°C for 7 min. After subsequent treatments of PCR products and the second PCR, Illumina sequencing was performed.

### Amplicon sequence data processing.

Amplicon sequence data were processed by the following procedure. Raw paired-end reads were merged using PEAR v0.9.10 (126), and the primer sequences were removed using Cutadapt v1.10 (127). Low-quality (Q score of <30 in over 3% of sequences) and short (<150 bp) reads were filtered out using a custom Perl script. The number of clean read sequences for each sample was 56,524 to 66,543 for 16S rRNA gene amplicons and 131,802 to 359,192 for *pmoA* amplicons. The resulting sequences were analyzed using the QIIME 2 v2019.4.0 pipeline (128). ASVs were generated using the DADA2 plugin wrapped in QIIME 2 (129), where Illumina-sequenced amplicon errors were corrected, and then chimeric sequences were removed. The taxa were assigned to the ASVs for 16S rRNA genes using the QIIME 2 plugin feature-classifier classify-sklearn (130) against the SILVA 132 database (131) and to the ASVs for *pmoA* using a BLASTX search of the NCBI nonredundant (nr) database. To assess the ASVs for *pmoA*, the ASVs were translated into protein sequences using a custom Perl script, and then those sequences were searched against the Pfam database (http://pfam.xfam.org/) using hmmscan v3.0 in HMMER (https://www.ebi.ac.uk/Tools/hmmer/).

### Amplicon sequence data analyses.

A neighbor-joining (NJ) tree for the ASVs was built using BIONJ (132) after alignment with MUSCLE v3.8.31 (133). Species-like groups of *pmoA* were generated based on the NJ tree of ASVs by a distance threshold of 0.04 (Jukes-Cantor distance). Alpha diversity analyses (Chao1, Shannon, and Simpson diversity indices) for species-like groups were performed using phyloseq v.1.30.0 (134) and vegan v.2.5.6 (135) packages in the R environment (http://www.R-project.org). A statistical comparison of the alpha diversity between the ISCSs was done using the Kruskal-Wallis test followed by Dunn’s *post hoc* test with Bonferroni correction of significance. The significance level was set at 0.05. Beta diversity was visualized by nonmetric multidimensional scaling (NMDS), in which the dissimilarity between samples was estimated with a Bray-Curtis distance matrix using the metaMDS and ordiellipse functions in the R package vegan (135).

### Genome reconstruction and gene annotation for a methanotroph in the cultivated biofilm.

For long-read sequencing of DNA from the methR-40 biofilm sample, DNA fragments larger than 5 kb were prepared using a BluePippin system (Sage Sciences, Beverly, MA, USA), and a PacBio SMRTbell library was constructed according to the manufacturer’s instruction. Sequencing on the PacBio Sequel platform (Pacific Biosciences, Menlo Park, CA, USA) yielded a total of 9.56 GB of sequences with an *N*_50_ length of 6.85 kb. The *de novo* assembly was performed using the HGAP4 (136) pipeline from the PacBio single-molecule real-time (SMRT) toolkit (SMRT link v6.0.0). The resulting assembly contained 310 contigs in a total of 13.87 Mb. From the metagenomic assembly, two circular contigs showing approximately equal and even sequence coverages (483×) were recognized as a chromosome and a plasmid of a single methanotroph, designated INp10. The contigs were error-corrected using Pilon v1.18 (137) with Illumina reads. The completeness and contamination of the genome were calculated with CheckM (138) using a set of lineage-specific genes of *Gammaproteobacteria*.

The coding sequences were identified using a combination of MetaGeneMark (139) and Glimmer-MG (140). The deduced amino acid sequences were subjected to a BLASTP search against an NCBI nr protein database, and their functional annotations were assigned manually based on the Kyoto Encyclopedia of Genes and Genomes (KEGG) Orthology (KO) database. The prediction of genes for rRNA, tRNA, and other noncoding RNAs was performed with Barrnap v0.9 (https://github.com/tseemann/barrnap), tRNAscan-SE v1.3.1 (141), and Rfam v13.0 (142), respectively.

### Genome reconstruction of *B. japonicus* and *B*. *platifrons* endosymbionts.

The genome sequence of the *B*. *platifrons* endosymbiont based on Illumina short reads was reported previously by Takishita et al. (44), members of this research group. The endosymbiont population within a single host individual has presented high heterogeneity, meaning the coexistence of very closely related but not identical strains. This heterogeneity has made genome reconstruction difficult due to assembly fragmentation; hence, many ambiguous regions have remained in the endosymbiont genome. Thus, in this study, to retrieve a higher-quality genome as a reference for the INp10 genome, whole-genome sequencing using the PacBio Sequel platform was performed on the endosymbionts of *B. japonicus* and *B*. *platifrons* collected from the off Hatsushima seep in Sagami Bay. The genomes were reconstructed based on long-read sequences after procedures that eliminate uncertainty due to heterogeneity as much as possible. Sequencing and assembly procedures are described in the supplemental materials. The gene annotation of the composite genomes of the endosymbionts was performed as described above for the cultivated methanotroph.

### Comparison of genomes and phylogenomic analysis.

The ANI and AAI among four *Methyloprofundus* genome sequences were calculated using OrthoANI (143) and the AAI calculator (http://enve-omics.ce.gatech.edu/aai/), respectively. To reconstruct a phylogenomic tree, amino acid sequences of 30 single-copy marker genes were extracted from proteomes of the genomes of 4 *Methyloprofundus* species and 20 reference strains. Multiple-sequence alignment was performed using MAFFT v7.312 (144), and ambiguously aligned positions were removed using trimAl v1.2 (145). After the alignment was concatenated, a maximum likelihood phylogenomic tree was inferred by RAxML v8.2.9 (146) with the LG4X+G model and 100 bootstrap replications. A best-fit amino acid substitution model was chosen using Aminosan v1.0 (147).

### RNA-seq library construction and sequencing.

For the cultivated biofilm samples, two kinds of transcriptome sequencing (RNA-seq) libraries were constructed. Total RNA-seq libraries were constructed using the Ion total RNA-seq kit v2 (Life Technologies) and sequenced on an Ion 318 chip using the Ion Torrent PGM platform (Life Technologies). For transcriptome analysis, rRNA-depleted RNA was prepared using a Ribo-Zero rRNA removal kit for Gram-negative bacteria (Epicentre Biotechnologies, Madison, WI, USA), and RNA-seq libraries were constructed. Sequencing was performed on an Ion 540 chip using the Ion Torrent Ion S5XL platform (Life Technologies).

For *B. japonicus* and *B*. *platifrons* endosymbionts, RNA-seq libraries were constructed with rRNA-depleted RNA prepared using both the RiboMinus eukaryote kit (Thermo Fisher Scientific) and Ribo-Zero rRNA removal kit for Gram-negative bacteria. Sequencing was performed in the same manner as that of the biofilm samples.

### Community analysis based on rRNA.

For cultivated biofilm community analysis, single-end reads from total RNA-seq libraries were trimmed and filtered using PRINSEQ v0.20.4 (148) with the following parameters: -trim_qual_left 20, -trim_qual_right 20, and -min_len 100. Reads were corrected using Pollux v1.00 (149) for substitutions, insertions, deletions, and homopolymers. The corrected reads were subjected to EMIRGE v0.61.0 (150) to assess the biofilm community structure based on 16S rRNA. Near full-length 16S rRNA sequences were reconstructed with a templated assembly approach. An EMIRGE reference database was created from the SILVA 132 SSU Ref NR99 database with taxonomic labels. Relative abundances of taxa were estimated based on normalized priors from the final EMIRGE iteration.

### Transcriptome analysis.

Sequencing of RNA-seq libraries constructed using rRNA-depleted RNA generated 18.7 to 22.7 million single-end reads from each biofilm sample and 74 million of those from the gill tissues of both mussels. Raw reads were trimmed and filtered using PRINSEQ v0.20.4 with the following parameters: -trim_qual_left 20, -trim_qual_right 20, and -min_len 100. The cleaned reads were aligned to the respective methanotroph genome assemblies and quantified using Rockhopper v2.03 (151, 152), which was specifically designed for the bacterial gene structures and transcriptomes. After this process, the aligned read counts were 8.7 to 11.7 million (38.3% to 62.5% of the total clean reads) for the INp10 genome, 7.4 million (10.0%) for the *B. japonicus* endosymbiont, and 6.0 million (8.2%) for the *B*. *platifrons* endosymbiont (Fig. S5). The aligned read counts were normalized with upper quartile normalization (153), and transcripts per million (TPM) values were calculated for genes. To compare transcriptome profiles among these methanotrophs, gene transcription levels were adjusted by the inverse percentile rank transformation. Transcription levels of 53 ribosomal protein genes were compared between biofilms and assessed using the Friedman test followed by a one-sided Wilcoxon signed-rank test with Bonferroni correction for pairwise comparisons. The significance level was set at 0.05.

### Data availability.

The 16S rRNA gene amplicon sequence data from the ISCSs and the enriched biofilms are available in the DNA Data Bank of Japan (DDBJ) Sequenced Read Archive (DRA; https://www.ddbj.nig.ac.jp/dra/index-e.html) under accession numbers DRA007525 and DRA007523, respectively. The *pmoA* gene amplicon sequence data from the ISCSs are available in the DRA under accession numbers DRA007526 and DRA012412. The metagenomic sequencing data are available in the DRA under accession numbers DRA009836 for the methR-40d biofilm, DRA009834 for the *B. japonicus* endosymbiont, and DRA009835 for the *B*. *platifrons* endosymbiont. The transcriptomic sequencing data are available in the DRA under accession numbers DRA009839 for the biofilms, DRA009837 for the *B. japonicus* endosymbiont, and DRA009838 for the *B*. *platifrons* endosymbiont. The genome sequences were deposited in DDBJ/EMBL/GenBank under the following accession numbers: AP023240 and AP023241 for *Methyloprofundus* sp. INp10; BLYC01000001 to BLYC01000109 for the *B. japonicus* endosymbiont; and BLYD01000001 to BLYD01000327 for the *B*. *platifrons* endosymbiont.
