# Supplementary material for: Multispecies Populations of Methanotrophic Methyloprofundus and Cultivation of a Likely Dominant Species from the Iheya North Deep-Sea Hydrothermal Field
Source: Appl Environ Microbiol. 2022 Jan 25;88(2):e00758-21. doi: 10.1128/AEM.00758-21 (PMC8788690; doi:10.1128/AEM.00758-21)
Supplement: Supplemental file 1 — Supplemental Materials and Methods, Tables S1 to S3 and S5 to S8, Fig. S1 to S6. Download AEM.00758-21-s0001.pdf, PDF file, 3.9 MB [file aem.00758-21-s0001.pdf]

# Supplemental Materials

## Materials and Methods

### Genome analysis of the methanotrophic endosymbionts of *B. japonicus* and "*B.*" *platifrons*

Genome sequencing for the methanotrophic endosymbionts of *B. japonicus* and "*B.*" *platifrons* was performed using DNA extracted from the gill tissues of the mussels. For long-read sequencing, DNA fragments larger than 5 kb were prepared using a BluePippin system (Sage Sciences), and a PacBio SMRTbell library was then constructed according to the manufacturer's instruction. Sequencing on the PacBio Sequel platform (Pacific Biosciences) yielded a total of 13.62 Gb sequences with an N50 length of 7.2 Kb for the *B. japonicus* symbiont, while a total of 10.64 Gb sequences with an N50 length of 8.9 Kb for the "*B.*" *platifrons* symbiont. The PacBio subreads were assembled using the HGAP4 (2) pipeline from the PacBio SMRT toolkit (SMRT Link v6.0.0) and Flye v2.5 assembler (3), independently. The genome was re-sequenced using the Illumina MiSeq platform to resolve the error found in SMRT sequencing. A paired-end library (insert size: 350 bp) was constructed with the KAPA library prep kit (Kapa Biosystems). MiSeq sequencing yielded a total of 0.53 Gb sequences for the *B. japonicus* symbiont and 8.9 Gb sequences for the "*B.*" *platifrons* symbiont. The Illumina raw reads were subjected to adaptor clipping and quality trimming using TRIMMOMATIC v0.36 (4) and error correction using Pilon v1.18 (5).

For the *B. japonicus* symbiont, two *de novo* assemblies, HGAP4 and Flye, reconstructed 631 contigs (total length: 15.7 Mb, and N50 size: 27.6 Kb) and 752 contigs (total length: 14.9 Mb, and N50 size: 37.8 Kb), respectively. For the "*B.*" *platifrons* symbiont, two *de novo* assemblies, HGAP4 and Flye, reconstructed 711 contigs (total length: 18.4 Mb, and N50 size: 31.3 Kb) and 1,832 contigs (total length: 27.5 Mb, and N50 size: 22.9 Kb), respectively. Due to larger N50 sizes, Flye assembly for the *B. japonicus* symbiont and HGAP4

assembly for the "*B.*" *platifrons* symbiont were selected as the respective primary assemblies. The final contigs were generated from the primary assembly and unique sequences that were included in the secondary assembly but not in the primary assembly. The contigs were error-corrected using Pilon v1.18 with Illumina reads. Redundant contigs in each assembly were removed using the only reduction step in the Redundans pipeline (6).

Subsequently, metagenomic binning of the assemblies from both symbionts was performed using MyCC (7) with default parameters. Low-quality contigs with <3 kb length, or <30× average coverage were discarded from the binning. The two-dimensional scatter plots in MyCC binning showed that non-redundant contigs were mainly separated into two genomic bins, BIN1 and BIN2. BIN1 contained many genes affiliated to the order *Methylococcales*, whereas BIN2 contained genes from the order *Campylobacterales*. Although it was inferred that BIN1 was composed of contigs from the methanotrophic endosymbiont, the contigs showed high structural heterogeneity, including variations in gene copy number, and the presence/absence of genes and insertion sequence elements. Then, redundant contigs with similar gene arrangements were manually removed, and the remaining contigs were manually combined to create a single contig that spans the genome of the endosymbiont as far as possible.

The above process was performed in four successive steps. First, the non-redundant gene catalog was obtained by clustering the coding sequences at 97% identity with CD-HIT v4.6.8 (8). Second, redundant contigs with similar gene arrangements were manually detected, and their sequence similarity was checked by pairwise alignment using MUMmer v3.23 (9). Third, the redundant contigs with a shorter length or lower coverage were removed. Finally, a composite genome of the methanotrophic endosymbiont of each of *B. japonicus* and "*B.*" *platifrons* was obtained (Table S1). The completeness and contamination of the genomes were calculated with CheckM (10) using a set of lineage-specific genes of *Gammaproteobacteria*.

# Supplementary Tables S1–S3, S5–S8

**TABLE S1** Genome sequence information of the *Methyloprofundus* species

| Data | INp10 | *M. sedimenti* WF1 | Endosymbiont of *B. japonicus* | Endosymbiont of "*B.*" *platifrons* |
|---|---|---|---|---|
| **BacBio Sequel sequencing data** | | | | |
| Total subreads (Million) | 1.73 | | 2.30 | 1.62 |
| Total size (Gp) | 9.55 | | 13.62 | 10.64 |
| **Assembly data** | | | | |
| Status | Complete | Draft | Draft | Draft |
| Genome size (bp) | 4,386,347 (+ 42,044)[a] | 4,290,526 | 4,971,076 | 6,592,405 |
| Number of contigs | Chromosome, 1 Plasmid,1 | 6 | 109 | 327 |
| Maximum contig length (Mb) | 4.39 | 2.90 | 0.49 | 0.14 |
| N50 (Mb) | 4.39 | 2.90 | 0.10 | 0.03 |
| CheckM_completeness (%) | 99.7 | 98.9 | 96.0 | 98.5 |
| CheckM_contamination (%) | 1.21 | 2.67 | 1.38 | 4.14 |
| Coverage (×) | 483 | 10 | 880 | 120 |
| G+C content (mol %) | 39.9 | 41.0 | 41.8 | 40.3 |
| **Gene data** | | | | |
| Protein coding genes | 3,774 | 3,699 | 2,869 | 4,160 |
| Pseudogene | 95 | 52 | 1,117 | 1,465 |
| rRNA | 9 | 9 | 9 | 9 |
| tRNA | 43 | 42 | 43 | 45 |
| ncRNA | 3 | 3 | 3 | 3 |
| tmRNA | 1 | 2 | 1 | 1 |
| Gene coding density (%)[b] | 86.3 | 80.7 | 53.4 | 54.8 |
| **GeneBank accession** | AP023240, AP023241 | LPUF01000001–LPUF01000006 | BLYC01000001–BLYC01000109 | BLYD01000001–BLYD01000327 |
| **Reference** | This study | (11) | This study | This study |

[a]Plasmid size is shown in parentheses.

[b]Gene coding density does not contain pseudogenes.

**TABLE S2** Single-copy marker genes used for a phylogenomic tree reconstruction

| No. | KEGG Orthology ID | Gene | Description |
|---|---|---|---|
| 1 | K00927 | pgk | phosphoglycerate kinase |
| 2 | K01937 | pyrG | CTP synthase |
| 3 | K02316 | dnaG | DNA primase |
| 4 | K02357 | tsf | elongation factor Ts |
| 5 | K02600 | nusA | transcription termination/antitermination protein NusA |
| 6 | K02838 | frr | ribosome recycling factor |
| 7 | K02863 | rplA | large subunit ribosomal protein L1 |
| 8 | K02867 | rplK | large subunit ribosomal protein L11 |
| 9 | K02871 | rplM | large subunit ribosomal protein L13 |
| 10 | K02874 | rplN | large subunit ribosomal protein L14 |
| 11 | K02878 | rplP | large subunit ribosomal protein L16 |
| 12 | K02884 | rplS | large subunit ribosomal protein L19 |
| 13 | K02886 | rplB | large subunit ribosomal protein L2 |
| 14 | K02887 | rplT | large subunit ribosomal protein L20 |
| 15 | K02899 | rpmA | large subunit ribosomal protein L27 |
| 16 | K02906 | rplC | large subunit ribosomal protein L3 |
| 17 | K02926 | rplD | large subunit ribosomal protein L4 |
| 18 | K02931 | rplE | large subunit ribosomal protein L5 |
| 19 | K02933 | rplF | large subunit ribosomal protein L6 |
| 20 | K02935 | rplL | large subunit ribosomal protein L7/L12 |
| 21 | K02946 | rpsJ | small subunit ribosomal protein S10 |
| 22 | K02948 | rpsK | small subunit ribosomal protein S11 |
| 23 | K02952 | rpsM | small subunit ribosomal protein S13 |
| 24 | K02965 | rpsS | small subunit ribosomal protein S19 |
| 25 | K02967 | rpsB | small subunit ribosomal protein S2 |
| 26 | K02982 | rpsC | subunit ribosomal protein S3 |
| 27 | K02988 | rpsE | small subunit ribosomal protein S5 |
| 28 | K02996 | rpsI | small subunit ribosomal protein S9 |
| 29 | K03043 | rpoB | DNA-directed RNA polymerase subunit beta |
| 30 | K03664 | smpB | SsrA-binding protein |

**TABLE S3** Summary of transcriptome data for the biofilms and the gill tissues of bathymodiolin mussels

| Data | Biofilm sample | | | *B. japonicus* gill tissue | *"B." platifrons* gill tissue |
|---|---|---|---|---|---|
| | methR-17d | methR-40d | methR-88d | | |
| **Ion S5XL sequencing data** | | | | | |
| Total number of raw reads | 23,641,628 | 20,566,640 | 19,855,762 | 78,935,274 | 79,992,771 |
| Total number of clean reads | 22,722,258 | 19,785,140 | 18,744,452 | 74,019,616 | 73,967,455 |
| % of clean reads to raw reads | 96.1% | 96.2% | 94.4% | 93.8% | 92.5% |
| Average length of reads (bp) | 119 | 141 | 140 | 167 | 163 |
| **Alignment to a reference methanotroph genome** | | | | | |
| Total number of aligned reads | 8,706,351 | 9,231,461 | 11,714,248 | 7,386,319 | 6,033,089 |
| % of aligned reads to clean reads | 38.3% | 46.7% | 62.5% | 10.0% | 8.2% |
| **Percentage of each gene in the aligned reads (%)** | | | | | |
| rRNA | 0.5 | 0.3 | 0.1 | <0.1 | <0.1 |
| tmRNA | 51.3 | 64.9 | 68.3 | 27.8 | 38.4 |
| ncRNA | 8.1 | 8.0 | 3.2 | 21.7 | 23.3 |
| Coding regions | 33.5 | 22.9 | 24.0 | 32.6 | 25.3 |
| Others | 5.5 | 3.6 | 4.1 | 13.2 | 9.7 |

**TABLE S5** Classification of gene transcriptions presented by TPM values

| Transcription level | Ranking category for TPM value | The minimum TPM value for each ranking category in: | | | | |
|---|---|---|---|---|---|---|
| | | INp10 in biofilm of: | | | *B. japonicus* symbiont | *"B." platifrons* symbiont |
| | | methR-17d | methR-40d | methR-88d | | |
| Extremely high | Top >1% | 2,834 | 2,472 | 2,204 | 4,008 | 2,526 |
| Very high | Top >3% | 1001 | 787 | 744 | 1,577 | 1,170 |
| | Top >5% | 628 | 504 | 451 | 1,113 | 775 |
| High | Top >10% | 306 | 252 | 243 | 627 | 422 |
| | Top >15% | 204 | 167 | 164 | 462 | 308 |
| Moderate | Top >30% | 108 | 88 | 88 | 243 | 154 |
| Low | Top >50% | 63 | 50 | 50 | 120 | 76 |
| Very low | Bottom ≦50% | 1 | 1 | 0 | 0 | 0 |

**TABLE S6** Summary of samples and experiments performed in this study

| Sample type | Sample ID | Experiment[a] | | | | | | | | | | Depth (m) | Sampling field[c] | Cruise ID (Dive No.) |
|---|---|---|---|---|---|---|---|---|---|---|---|---|---|---|
| | | Cultivation | 16S rRNA gene amplicon | rRNA composition | *pmoA* amplicon | Meta-Genome | Meta-Transcriptome | FISH | SEM | TEM | PCFM[b] | | | |
| ISCS | ISCS-1 | | ✓ (DRR155417) | | ✓ (DRR155421, DRR308206, DRR308207) | | | ✓ | | | ✓ | 1,058 | Iheya North, MOT | NT13-22 (HPD#1592), KY14-01 (HPD#1613) |
| ISCS | ISCS-2 | ✓ Inoculum | ✓ (DRR155418) | | ✓ (DRR155422, DRR308208, DRR308209) | | | | | | ✓ | 1,061 | Iheya North, MOT | NT13-22 (HPD#1593), KY14-01 (HPD#1619) |
| ISCS | ISCS-3 | ✓ Inoculum | ✓ (DRR155419) | | ✓ (DRR155423, DRR308210, DRR308211) | | | ✓ | ✓ | | ✓ | 994 | Iheya North, MOT | NT13-22 (HPD#1593), KY14-01 (HPD#1610) |
| ISCS | ISCS-4 | | ✓ (DRR155420) | | ✓ (DRR155424, DRR308212, DRR308213) | | | ✓ | | | ✓ | 986 | Iheya North, MOT | NT13-22 (HPD#1593), KY14-01 (HPD#1610) |
| *B. japonicus*, gill tissue | | ✓ Inoculum | | | | | | | | | ✓ | 986 | Iheya North, MOT | NT09-17 (HPD#1062) |
| cultivated biofilm | methR-17d | | ✓ (DRR155411) | ✓ (DRR155414) | | | ✓ (DRR216667) | | | | | | | |
| cultivated biofilm | methR-40d | | ✓ (DRR155412) | ✓ (DRR155415) | | ✓ (DRR216660, DRR216661) | ✓ (DRR216668) | | | | | | | |
| cultivated biofilm | methR-88d | | ✓ (DRR155413) | ✓ (DRR155416) | | | ✓ (DRR216669) | | | | | | | |
| cultivated biofilm | | | | | | | | ✓ | ✓ | ✓ | ✓ | | | |
| *B. japonicus*, gill tissue | | ✓ Inoculum | | | | | | | | | ✓ | 1,023 | Iheya North, MOT | NT10-E01 (HPD#1178) |
| *B. japonicus*, gill tissue | HPD1508-BjaS1 | | | | | ✓ (DRR216656, DRR236244) | | | | | | 857 | Off Hatsushima, Sagami Bay | NT13-07 (HPD#1508) |
| "*B.*" *platifrons*, gill tissue | HPD1508-BplS1 | | | | | ✓ (DRR216658, DRR216659) | | | | | | 857 | Off Hatsushima, Sagami Bay | NT13-07 (HPD#1508) |
| *B. japonicus*, gill tissue | HPD1643-BjaS1 | | | | | | ✓ (DRR216662) | | | | | 957 | Off Hatsushima, Sagami Bay | NT14-05 (HPD#1643) |
| "*B.*" *platifrons*, gill tissue | HPD1643-BplS1 | | | | | | ✓ (DRR216665) | | | | | 957 | Off Hatsushima, Sagami Bay | NT14-05 (HPD#1643) |

[a]Run accession numbers for sequence data are shown in parentheses.

[b]PCFM, phase contrast/fluorescence microscopy

[c]MOT, mid-Okinawa Trough

**TABLE S7** Mp731 probe specificity to the taxonomic groups within the order *Methylococcales*

| Taxonomic group[a] or strain | Sequence data | Match/Mismatch to Mp731[b] |
|---|---|---|
| *Methyloprofundus* | SILVA database | 100% match, 93% (53/57)[c] |
| *Methyloprofundus* | ASVs from 16S rRNA gene amplicon analysis | 100% match, 100% (17/17) |
| MMG2 | SILVA database | 1-base mismatch, 7% (5/71)<br>≧2-base mismatch, 93% (66/71) |
| MMG2 | ASVs from 16S rRNA gene amplicon analysis | 1-base mismatch, 23% (5/22)<br>≧2-base mismatch, 77% (17/22) |
| pLW-20 | SILVA database | ≧2-base mismatch, 100% (21/21) |
| pLW-20 | ASVs from 16S rRNA gene amplicon analysis | ≧2-base mismatch, 100% (10/10) |
| Milano-WF1B-42 | SILVA database | 100% match, 100% (3/3)[d] |
| *Methylobacter marinus* MR1 (negative control strain) | Sequence obtained in our laboratory | 2-base mismatch |

[a]Taxonomic group names used in the SILVA database

[b]The number of corresponding sequences to the total number of sequences is shown in parentheses.

[c]The sequences of INp10 and the "*B.*" *platifrons* endosymbiont, which are not included in the SILVA database, are included.

[d]Milano-WF1B-42 is not involved in this study, but it was the sole group showing 100% match with Mp731 other than *Methyloprofundus* in the SILVA database.

**TABLE S8** Primers and probes used in this study

| Primer and Probe | Sequence (5' to 3') : (mix ratio) | Target (positions[a]) | Reference |
|---|---|---|---|
| **PCR primer** | | | |
| MPFpmo-F mix | | *pmoA* (184–203) | This study |
| MPFpmo-F1 | ACTGTAGCGCCAATCGTATC: (1) | | |
| MPFpmo-F2 | ACTGTAGCGCCGATCGTTTC: (1) | | |
| MPFpmo-R | ACTGGAGCAACRTCTTTACC | *pmoA* (625–644) | This study |
| F515 | GTGCCAGCMGCCGCGGTAA | 16S rRNA gene (515–533) | (12) |
| R806 | GGACTACHVGGGTWTCTAAT | 16S rRNA gene (787–806) | (12) |
| U530F mix | | 16S rRNA gene (515–530) | (13) |
| Bac 530F | GTGCCAGCAGCCGCGG: (30) | | |
| Arch 530F | GTGBCAGCCGCCGCGG: (24) | | |
| Arch2 530F | YTGCCAGCCGCCGCGG: (6) | | |
| Bac2 530F | GTGCCAGCAGCWGCGG: (1) | | |
| Bac3 530F | GTGCCAGCAGTCGCGG: (1) | | |
| Bac4 530F | GTGCCAGAAGMMTCGG: (1) | | |
| Nano 530F | GTGGCAGTCGCCACGG: (3) | | |
| U907R mix | | 16S rRNA gene (907–926) | (13) |
| Uni 907R | CCGYCAATTCMTTTRAGTTT: (20) | | |
| DeepAB 907R | CCGYCTATTCCTTTGAGTTT: (1) | | |
| SAG-Del 907R | CCGYCAATTTCTTTRAGTTT: (1) | | |
| DeepAB2 907R | CCGYCAATTCCCTTRAGTTT: (1) | | |
| Arch2 907R | CCGYCAATTCCTTMAAGTTT: (1) | | |
| OP11 907R | CCGCCAATTCCTTTGAATTT: (1) | | |
| **Sequences added to primers for Illumina amplicon sequencing** | | | |
| Illumina adaptor sequence | ACACTCTTTCCCTACACGACGCTCTTCCGATCT | | |
| Illumina Multiplexing PCR Primer 2.0 sequence | GTGACTGGAGTTCAGACGTGTGCTCTTCCGATCT | | |
| **FISH probe** | | | |
| Probe Mp731 | CAGTTTTAGTCCAGGAAGTCGC | 16S rRNA (731–752) | This study |
| Eub338 | GCTGCCTCCCGTAGGAGT | 16S rRNA (338–355) | (14) |
| NonEub | ACTCCTACGGGAGGCAGC | 16S rRNA | (15) |

[a]The target positions are indicated by those in *M. sedimenti* WF1$^T$ for the *pmoA* genes, and those in *E. coli* for the 16S rRNA (genes).

## References


1. Kuwahara H, Yoshida T, Takaki Y, Shimamura S, Nishi S, Harada M, Matsuyama K, Takishita K, Kawato M, Uematsu K, Fujiwara Y, Sato T, Kato C, Kitagawa M, Kato I, Maruyama T. 2007. Reduced genome of the thioautotrophic intracellular symbiont in a deep-sea clam, *Calyptogena okutanii*. Curr Biol 17:881-886.
2. Chin C-S, Alexander DH, Marks P, Klammer AA, Drake J, Heiner C, Clum A, Copeland A, Huddleston J, Eichler EE, Turner SW, Korlach J. 2013. Nonhybrid, finished microbial genome assemblies from long-read SMRT sequencing data. Nat Methods 10:563-569.
3. Kolmogorov M, Yuan J, Lin Y, Pevzner PA. 2019. Assembly of long, error-prone reads using repeat graphs. Nat Biotechnol 37:540-546.
4. Bolger AM, Lohse M, Usadel B. 2014. Trimmomatic: a flexible trimmer for Illumina sequence data. Bioinformatics 30:2114-2120.
5. Walker BJ, Abeel T, Shea T, Priest M, Abouelliel A, Sakthikumar S, Cuomo CA, Zeng Q, Wortman J, Young SK, Earl AM. 2014. Pilon: an integrated tool for comprehensive microbial variant detection and genome assembly improvement. PLoS One 9:e112963.
6. Pryszcz LP, Gabaldón T. 2016. Redundans: an assembly pipeline for highly heterozygous genomes. Nucleic Acids Res 44:e113.
7. Lin H-H, Liao Y-C. 2016. Accurate binning of metagenomic contigs via automated clustering sequences using information of genomic signatures and marker genes. Sci Rep 6:24175.
8. Li W, Godzik A. 2006. Cd-hit: a fast program for clustering and comparing large sets of protein or nucleotide sequences. Bioinformatics 22:1658-1659.
9. Kurtz S, Phillippy A, Delcher AL, Smoot M, Shumway M, Antonescu C, Salzberg SL. 2004. Versatile and open software for comparing large genomes. Genome Biol 5:R12.
10. Parks DH, Imelfort M, Skennerton CT, Hugenholtz P, Tyson GW. 2015. CheckM: assessing the quality of microbial genomes recovered from isolates, single cells, and metagenomes. Genome Res 25:1043-1055.
11. Tavormina PL, Kellermann MY, Antony CP, Tocheva EI, Dalleska NF, Jensen AJ, Valentine DL, Hinrichs K-U, Jensen GJ, Dubilier N, Orphan VJ. 2017. Starvation and recovery in the deep-sea methanotroph *Methyloprofundus sedimenti*. Mol Microbiol 103:242-252.
12. Caporaso JG, Lauber CL, Walters WA, Berg-Lyons D, Lozupone CA, Turnbaugh PJ, Fierer N, Knight R. 2011. Global patterns of 16S rRNA diversity at a depth of millions of sequences per sample. Proc Natl Acad Sci USA 108:4516-4522.
13. Nunoura T, Takaki Y, Kazama H, Hirai M, Ashi J, Imachi H, Takai K. 2012. Microbial diversity in deep-sea methane seep sediments presented by SSU rRNA gene tag sequencing. Microbes Environ 27:382-390.

14. Amann RI, Binder BJ, Olson RJ, Chisholm SW, Devereux R, Stahl DA. 1990. Combination of 16S rRNA-targeted oligonucleotide probes with flow cytometry for analyzing mixed microbial populations. Appl Environ Microbiol 56:1919-1925.
15. Wallner G, Amann R, Beisker W. 1993. Optimizing fluorescent in situ hybridization with rRNA-targeted oligonucleotide probes for flow cytometric identification of microorganisms. Cytometry 14:136-143.

## Supplementary Figures S1–S6

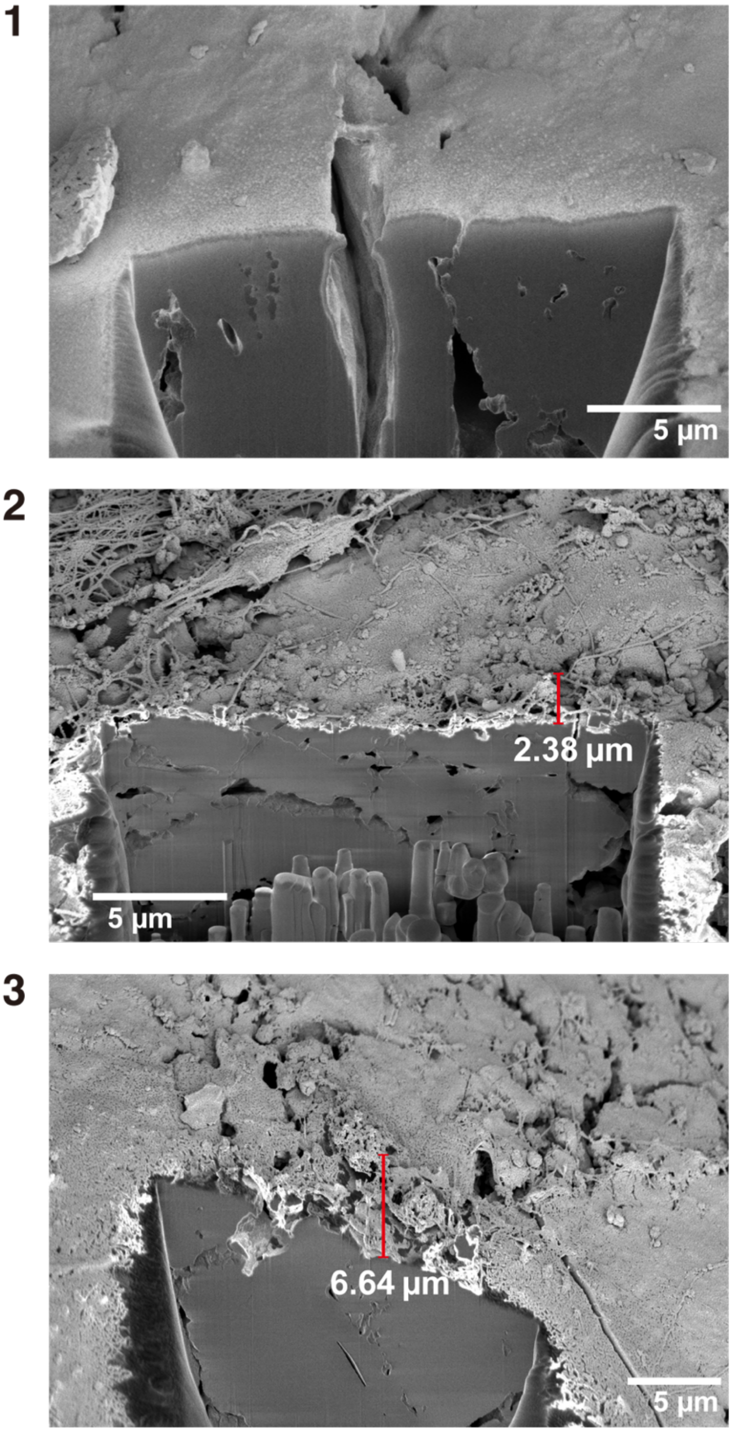

## FIG. S1

FIB-SEM micrographs of ceramic particles. **Panel 1** shows a ceramic particle before deployment, and **panels 2** and **3** show the particles in the ISCS-3 examined as a representative. To determine the thickness of biofilms formed on the particle surface, sample milling was performed with a gallium ion beam at 30 kV with a current of 1.2 to 9.1 nA.

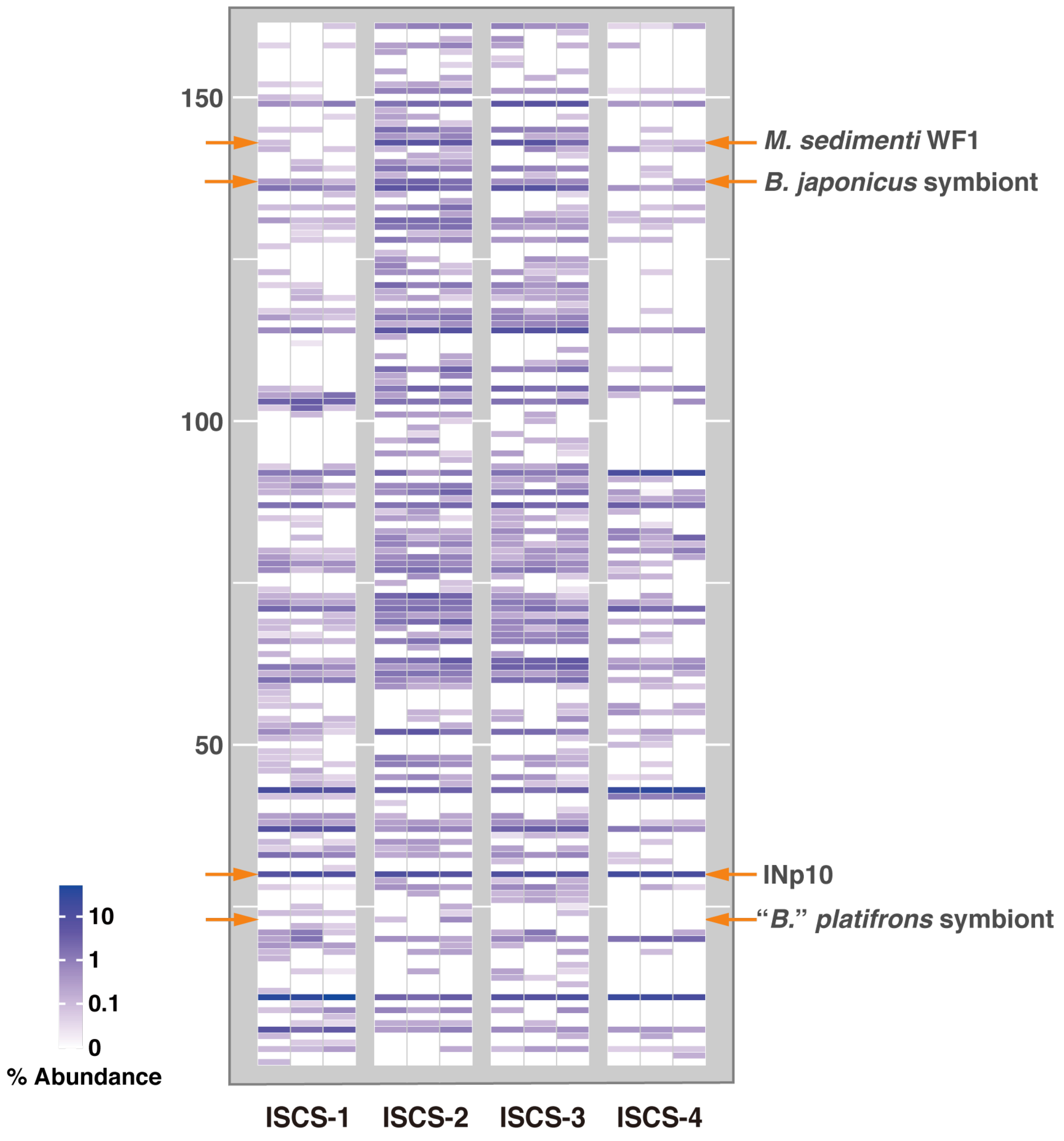


## FIG. S2

Heatmap showing the species-like group composition of the *Methyloprofundus* population in each sample of the ISCSs. A total of 161 species-like groups were identified by analyzing 50,000 reads of the *pmoA* amplicons of *Methyloprofundus* in each sample.

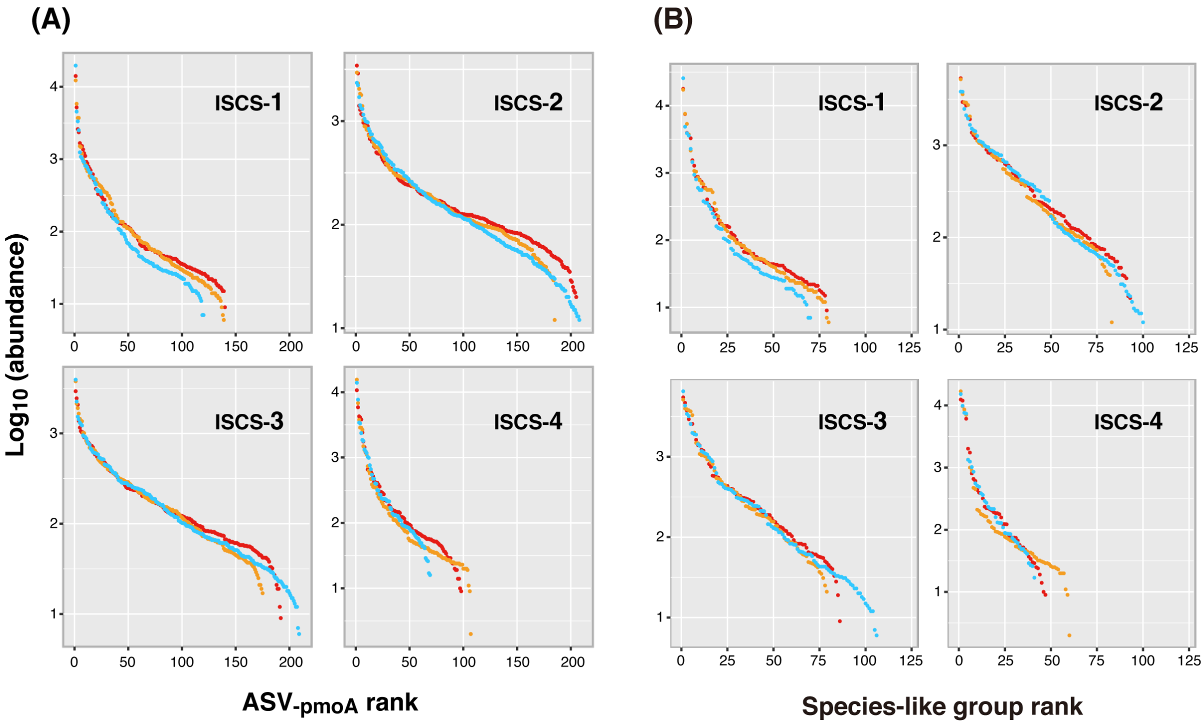


**FIG. S3**

Rank-abundance curves based on both ASVs of *pmoA* **(A)** and species-like groups **(B)** in the *Methyloprofundus* populations. Abundance shows the number of sequence reads. Different samples are shown in different colors.

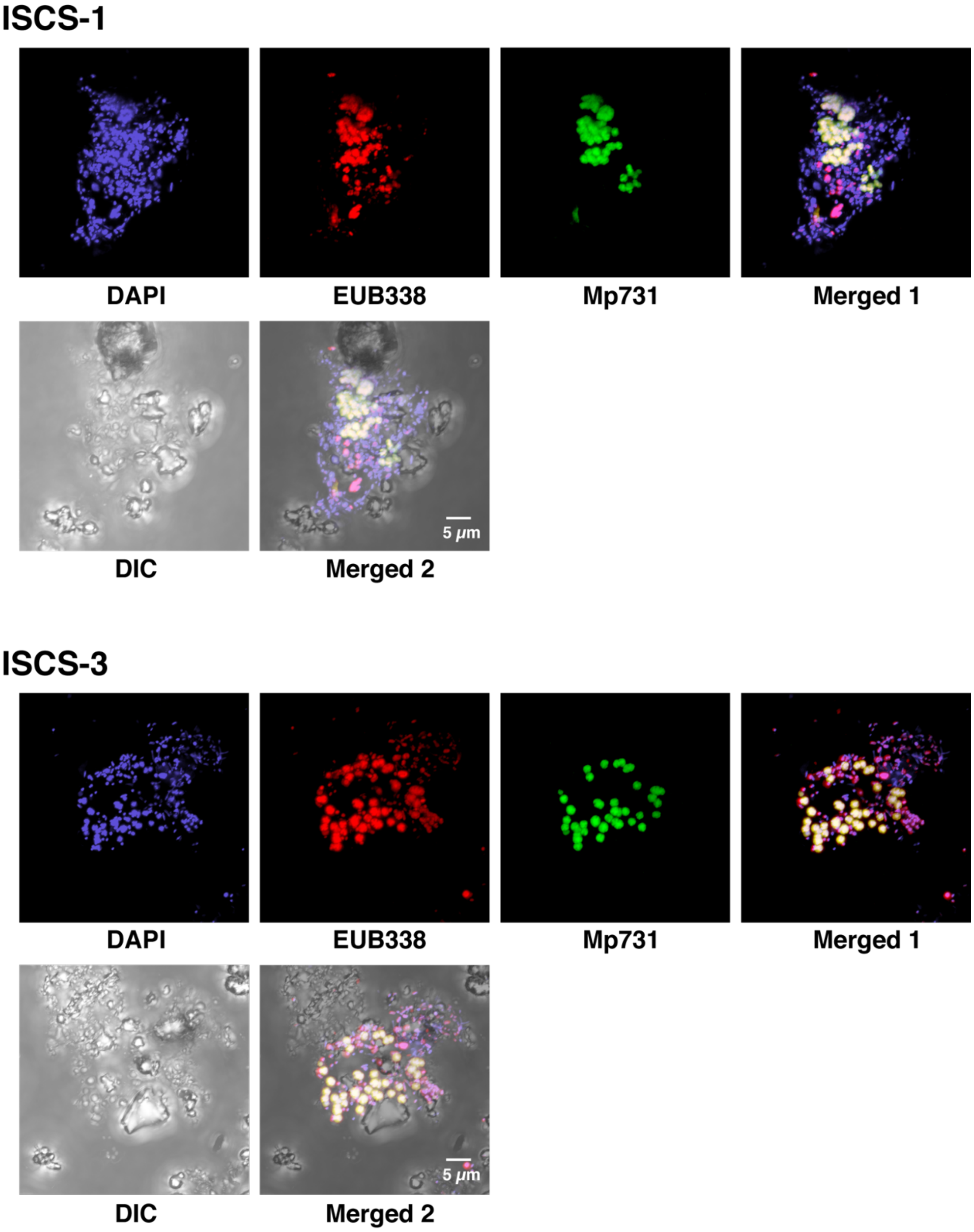


## FIG. S4

Whole-cell FISH images of *Methyloprofundus*-like bacteria colonized on ceramic particles of ISCS-1 and ISCS-3. The Mp731 probe was designed to detect the *Methyloprofundus* clade. The three images, DAPI staining and FISH with the EUB338 and Mp731 probes, were merged as Merged 1. The image by DIC microscopy was further merged as Merged 2. The MP731 and Eub338 probes were labeled with Alexa Flour 488 and 555, respectively.

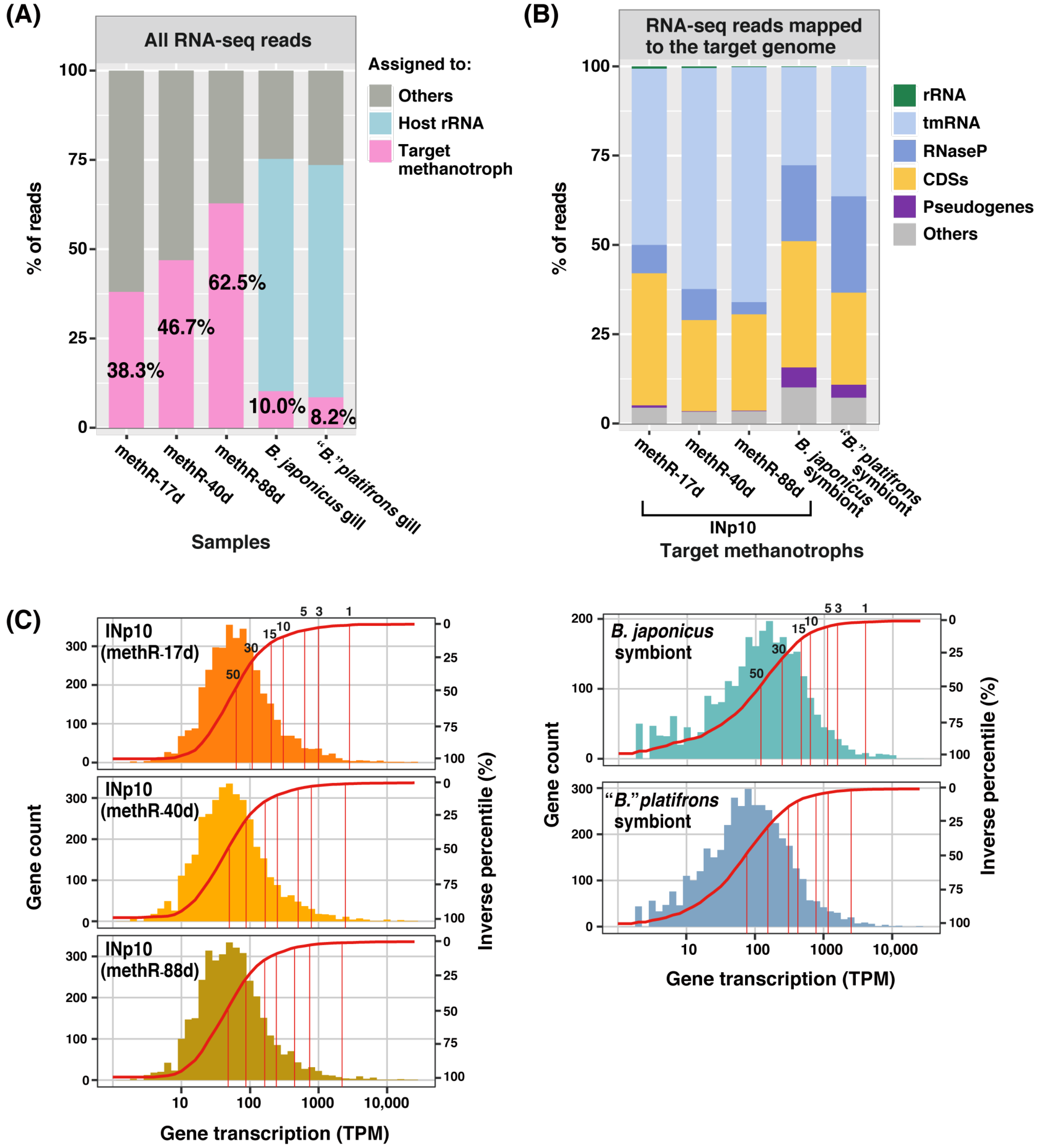


# FIG. S5

**(A)** The origin of all RNA-seq reads obtained from biofilms (methR-17d, methR-40d, and methR-88d) and gill tissues of *B. japonicus* and "*B.*" *platifrons*. **(B)** Properties of the RNA-seq reads mapped to the respective target methanotroph genomes. **(C)** A histogram showing gene distribution (left axis) and a line plot showing inverse percentile (1 – percentile) rank (right axis) based on TPM. Additional vertical lines indicate the points of inverse percentile rank to separate the transcriptional level categories (1%, 3%, 5%, 10%, 15%, 30%, and 50%).

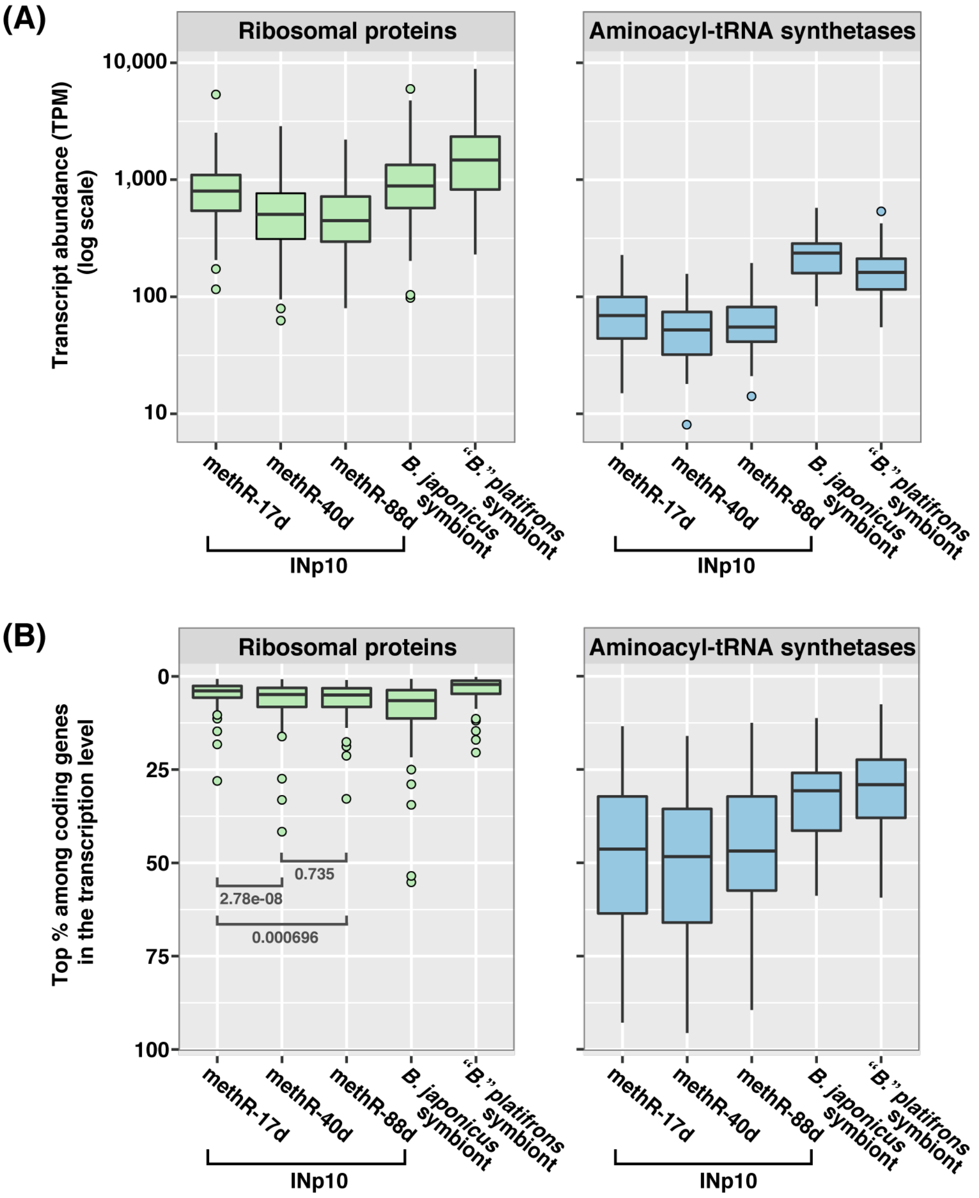


## FIG. S6

Transcript abundance **(A)** and top percentage (inverse percentile rank) among coding genes in the transcription level **(B)** for genes encoding ribosomal proteins and aminoacyl-tRNA synthetases in the target methanotroph genomes. To infer the metabolic activity change in INp10, the transcription rankings of ribosomal protein genes (53 genes) were compared between the biofilms using the Friedman test (a significant level, 0.05), and a statistically significant change (P = 2.24e–06) was observed. The following pairwise comparisons using a one-sided Wilcoxon signed-rank test with Bonferroni correction indicated that the rankings in methR-17d biofilm were significantly higher than in the other two biofilms (P values are indicated in the graph).
